# Association between perioperative optic nerve sheath diameter and postoperative delirium after Bentall surgery: a prospective observational study

**DOI:** 10.3389/fmed.2026.1940776

**Published:** 2026-09-15

**Authors:** Anıl Onur, Korgün Ökmen, Tuğba Onur, Ümran Karaca, Mehmet Ataseven, Sezgin Cecer, Asiye Demirel, Cihan Sedat Aytünür, Filiz Ata, Ayşe Neslihan Balkaya, Osman Sıla Aydın, Burak Erdolu, Arda Aybars Pala

**Affiliations:** 1 Department of Anesthesiology and Reanimation, University of Health Sciences, Bursa Yüksek İhtisas Training and Research Hospital, Bursa, Türkiye; 2 Department of Cardiovascular Surgery, University of Health Sciences, Bursa Yüksek İhtisas Training and Research Hospital, Bursa, Türkiye

**Keywords:** Bentall procedure, cardiopulmonary bypass, cerebral oximetry, optic nerve sheath diameter, perioperative neuromonitoring, postoperative delirium, ultrasonography

## Abstract

**Background:**

Optic nerve sheath diameter (ONSD) is an indirect, technique-dependent marker of intracranial pressure; its association with delirium after Bentall surgery remains uncertain. Therefore, we assessed serial perioperative ONSD and its relationship with delirium.

**Methods:**

This prospective single-centre study included 53 adults undergoing elective Bentall surgery. Delirium analyses included 51 participants whose status was assessable with the Confusion Assessment Method for the Intensive Care Unit; two remained unassessable until death. Bilateral ONSD was measured at 10 perioperative time points. Mixed-effects models evaluated serial ONSD, time-point comparisons were Holm-adjusted, and measures underwent Firth regression and receiver operating characteristic analyses. Sensitivity analyses assigned each indeterminate outcome as either delirium present or absent.

**Results:**

Documented delirium occurred in 17 of 51 participants with assessable delirium status (33.3%). Average perioperative ONSD was 0.250 mm higher with delirium (95% confidence interval 0.031–0.470; *p* = 0.025). Across outcome assignments, the difference ranged from 0.237 to 0.248 mm (*p*-values ≤ 0.029), whereas the time-by-group interaction was sensitive to assignment (*p*-values ranged from <0.001 to 0.094). No point-specific comparison survived Holm correction (smallest adjusted *p* = 0.078), and the post-closure T6 minus preoperative baseline showed no clear group difference (*p* = 0.147). In an exploratory Firth analysis not adjusted for time-point selection, the odds ratio per 1-mm higher T6 ONSD was 3.75 (95% confidence interval 1.44–12.32; *p* = 0.005). Areas under the receiver operating characteristic curves were 0.654 for Preop, 0.689 for T0, 0.728 for T6, and 0.626 for change, without evidence of superior T6 discrimination. The five-variable model had an apparent area under the curve of 0.839 and a conditional optimism-corrected value of 0.778; predictor and time-point selection were not repeated during validation. The internally selected 4.90-mm operating point was not an intracranial-hypertension threshold.

**Conclusions:**

Higher perioperative ONSD was associated with documented delirium, but the temporal interaction was sensitive to unobserved outcomes, and the data did not establish a superior time point or a causal intraoperative change. These findings require external validation.

**Trial registration:**

ClinicalTrials.gov NCT07452367; retrospectively registered 5 March 2026.

## Introduction

Postoperative delirium (POD) is a frequent form of acute brain dysfunction after cardiac surgery and is associated with longer hospitalisation and higher mortality; it is distinct from perioperative neurocognitive disorders, which are defined using different time windows and cognitive and functional criteria (1, 2). Patients undergoing Bentall and other complex aortic root procedures may be exposed to prolonged cardiopulmonary bypass (CPB), hypothermia, selective cerebral perfusion, and, in some cases, circulatory arrest. Neurocognitive deficits have been documented after deep hypothermic circulatory arrest, and perioperative neurological dysfunction has been associated with poorer long-term outcomes after aortic root replacement (3, 4). This setting is therefore relevant for evaluating practical perioperative markers of acute cerebral vulnerability.

The optic nerve sheath is continuous with the intracranial subarachnoid space, and its sonographic diameter has been studied as a non-invasive marker associated with raised intracranial pressure in neurocritical care populations (5–7). Nevertheless, ultrasound-derived optic nerve sheath diameter (ONSD) is an indirect, operator- and technique-dependent surrogate rather than a direct measurement of intracranial pressure, and no universal diagnostic threshold applies across populations and acquisition methods. Absolute ONSD may also be influenced by ocular anatomy and eyeball transverse diameter (8, 9). Although CPB has been associated with systemic inflammation and postoperative blood–brain barrier disruption, these mechanisms cannot be inferred from ONSD alone (10, 11). Accordingly, perioperative ONSD should be interpreted as an anatomical ultrasound measurement that may be influenced by intracranial dynamics, rather than as proof of cerebral oedema or intracranial hypertension.

Previous cardiac surgery studies have described ONSD changes during extracorporeal circulation and across perfusion strategies (12, 13). In a prospective open-heart surgery cohort, perioperative ONSD was associated with POD, whereas a carotid endarterectomy study linked a decrease in coronal ONSD with postoperative cognitive decline (14, 15). These studies differed in surgical population, acquisition technique, measurement schedule, and neurological outcome. Evidence specific to serial bilateral ONSD assessment during Bentall surgery remains limited, and it is uncertain whether an observed association with POD reflects pre-existing between-patient differences, intraoperative change, or both.

We therefore conducted a prospective observational study of adults undergoing elective Bentall surgery. The primary objective was to assess the association between perioperative bilateral ONSD and postoperative delirium. Secondary objectives were to describe serial bilateral ONSD and regional cerebral oxygen saturation trajectories and examine short-term change on the Montreal Cognitive Assessment (MoCA). Analyses comparing baseline, late intraoperative T6 after sternal closure, and change-from-baseline ONSD measures, as well as time-point selection, sample-derived cut-offs, and multivariable discrimination, were considered exploratory and require external validation.

## Materials and methods

### Study design, ethics, and participants

This prospective, single-centre observational study was conducted at the University of Health Sciences, Bursa Yüksek İhtisas Training and Research Hospital, a tertiary referral centre with a high-volume aortic surgery programme. The study complied with the Declaration of Helsinki and was approved by the Medical Sciences Research Ethics Committee of the institution (approval no. 2024-TBEK 2025/07-01; 30 July 2025). Written informed consent was obtained from all participants. The study was registered at ClinicalTrials.gov (NCT07452367; retrospectively registered on 5 March 2026) and is reported in accordance with the Strengthening the Reporting of Observational Studies in Epidemiology (STROBE) statement (16).

Consecutive adults aged 18–80 years who were scheduled for elective Bentall surgery between August 2025 and June 2026 were screened. All underwent elective composite aortic root replacement for an aortic root aneurysm with associated aortic valve disease. Patients were excluded for emergency surgery, known severe carotid stenosis (>70%), previous cerebrovascular disease or residual neurological sequelae, known cognitive impairment or dementia; a current intracranial lesion or mass, ocular or optic nerve disease or previous ocular surgery that could affect optic nerve sheath diameter (ONSD), antidepressant use or advanced psychiatric disease, or known advanced hepatic or renal failure. During the predefined recruitment period, 60 consecutive patients were assessed for eligibility. Five were excluded before enrolment because the investigator was unavailable (*n* = 2), consent was declined (*n* = 1), or the preoperative MoCA could not be completed validly (*n* = 2). Fifty-five patients were enrolled and underwent surgery. Two enrolled patients were excluded intraoperatively because they could not be separated from CPB, preventing completion of the perioperative measurement protocol; 53 patients therefore comprised the full study cohort. No *a priori* sample size or precision calculation was performed; enrolment covered the predefined 11-month recruitment period, and all inferential analyses were considered exploratory and hypothesis-generating.

No participant had a documented diagnosis of Marfan syndrome or another heritable thoracic aortic disease (0/53). This was based on clinical records and routine preoperative assessments; systematic assessment against the Ghent criteria (17), genetic testing, and detailed aetiological subclassification of the aortic root disease were not performed. Because no participant had a documented diagnosis, excluding documented cases did not alter either the full cohort or the population with assessable delirium status, and no separate estimate is shown.

### Outcomes and perioperative data

The primary outcome was in-hospital postoperative delirium detected with the Confusion Assessment Method for the Intensive Care Unit (CAM-ICU) from intensive care unit (ICU) admission until postoperative day 10, hospital discharge, or death, whichever occurred first. The primary analysis assessed its association with serial perioperative bilateral ONSD. Secondary outcomes were the perioperative trajectories of ONSD and regional cerebral oxygen saturation (rSO_2_), inter-eye ONSD concordance, early postoperative change in the MoCA score, the exploratory discrimination of selected ONSD and rSO_2_ measurements for delirium, and 10-day mortality. Mortality analyses were descriptive due to the small number of events.

The MoCA was included as an outcome measure in the study protocol. For an exploratory screening analysis, early postoperative MoCA change was defined as a decrease of at least two points from the preoperative score. The two-point threshold was a study-defined exploratory cut-off and was not interpreted as a diagnosis of delayed neurocognitive recovery or another perioperative neurocognitive disorder (2).

Recorded covariates included demographic and clinical characteristics, left ventricular ejection fraction, European System for Cardiac Operative Risk Evaluation II (EuroSCORE II), operative procedure, CPB and aortic cross-clamp durations, total circulatory arrest (TCA) and its duration, core temperature, intraoperative haemodynamic and blood-gas variables, fluid balance, transfusions, postoperative 0–10 numerical rating scale (NRS) pain scores at 6, 12, and 24 h, complications, and ICU and hospital lengths of stay. NRS was recorded only when a participant had been extubated and could communicate at the scheduled assessment; the corresponding available-case denominators were 24, 47, and 50 participants. Mean arterial pressure, arterial carbon dioxide tension (PaCO_2_), haematocrit, and pH were recorded at T0–T6; core temperature was recorded at all 10 study time points. Central venous pressure was monitored continuously to guide fluid management, but it was not entered on the case report form at the predefined time points and therefore could not be included in time-aligned analyses.

### Anaesthesia, surgery, and postoperative analgesia

No premedication was administered. Standard monitoring was supplemented by a bispectral index (BIS) sensor, bilateral cerebral oximetry sensors, radial arterial catheterisation, and central venous pressure monitoring. Anaesthesia was induced intravenously with midazolam 0.02–0.03 mg/kg, thiopental 3–5 mg/kg, fentanyl 2 µg/kg, and rocuronium 0.8 mg/kg, followed by tracheal intubation. Initial ventilation settings were an inspired oxygen fraction of 0.50, a tidal volume of 6 mL/kg, a positive end-expiratory pressure of 5 cmH_2_O, and a respiratory rate of 12/min. Anaesthesia was maintained with sevoflurane at 0.8–1.0 minimum alveolar concentration and intermittent fentanyl and rocuronium, targeting a BIS of 40–60 and a mean arterial pressure of 65–80 mmHg. The total intraoperative fentanyl dose was recorded in micrograms. All patients received a continuous glyceryl trinitrate infusion, titrated within the protocol limits of 0.2–2.0 µg/kg/min to the arterial pressure target. Patient-level infusion rates were not retained. Volatile anaesthetic maintenance continued during CPB.

Non-pulsatile CPB was used with a target pump flow corresponding to a cardiac index of 2.2–2.4 L/min/m^2^ and a mean arterial pressure of 65–80 mmHg. Haematocrit was maintained at 24%–32%, an alpha-stat strategy was used during CPB, and del Nido cardioplegia was used for myocardial protection (18). Cooling and antegrade or retrograde cerebral perfusion were selected according to surgical requirements. Core temperature was measured routinely with a bladder probe; nasopharyngeal temperature was used as a cross-check when the bladder reading was considered unreliable. Core temperature was recorded at each of the 10 study time points; the nadir core temperature, obtained at T3, was used to describe the degree of hypothermia.

The CPB circuit comprised a Stöckert S5 heart–lung machine (LivaNova/Sorin Group Deutschland GmbH, Munich, Germany) and an Inspire 8F hollow-fibre membrane oxygenator with an integrated arterial filter (Sorin Group Italia S.r.l., Mirandola, Italy). The prime contained 1,000 mL of Ringer's lactate, six ampoules of sodium bicarbonate, and 10,000 units of heparin. Conventional ultrafiltration with a Disposet FX600 haemoconcentrator (Disposet Medical Products, Ankara, Türkiye) was used when blood urea nitrogen exceeded 45 mg/dL or creatinine exceeded 1.5 mg/dL.

TCA was used in 26 of the 53 analysed patients; the remaining 27 underwent surgery without TCA. The TCA protocol comprised intravenous methylprednisolone 2 mg/kg, mannitol, and an additional thiopental sodium bolus of 5–7 mg/kg, and all 26 patients who underwent TCA received these agents. Methylprednisolone and mannitol were also recorded in 19 and 16 of the 27 patients without TCA, respectively. Administered mannitol volumes were recorded. A verified mannitol preparation concentration or dosing rule was unavailable, so no mass dose was inferred. No dedicated patient-level thiopental dose variable was retained. Inotropic and vasopressor therapy was administered as clinically indicated.

At the end of surgery, all patients received a bilateral ultrasound-guided parasternal fascial-plane block with 20 mL of 0.25% bupivacaine on each side (40 mL and 100 mg in total). Postoperative systemic analgesia consisted of intravenous paracetamol 10 mg/kg and tramadol 1 mg/kg, each administered twice daily. One patient received a single 100-mg dose of pethidine as rescue analgesia. Patients were weaned from mechanical ventilation without a postoperative sedative infusion. No postoperative benzodiazepine or dexmedetomidine was administered; midazolam was confined to induction. Residual effects of intraoperative anaesthetic and analgesic drugs were nevertheless possible at early postoperative assessments.

### ONSD and cerebral oximetry measurements

Bilateral ONSD was measured using an M5 portable ultrasound system (Mindray Bio-Medical Electronics, Shenzhen, China) and a 7L4s linear-array transducer at a nominal frequency of 7.5 MHz. The system had no dedicated ophthalmic preset; the small-parts preset was therefore used. Imaging was performed in B-mode only, and Doppler was not used. Standardised A-scan and the 30° test were unavailable and were not performed.

With the patient supine, the probe was applied transpalpebrally to the closed upper eyelid using ample gel and minimal pressure. In an axial plane displaying the optic nerve, calipers were placed to measure the sheath diameter 3 mm behind the globe and perpendicular to the nerve axis. Three acquisitions were obtained from each eye at every time point. Their arithmetic mean was used as the eye-level value, and the mean of the right- and left-eye values was the patient-level ONSD used in outcome analyses. Measurements were not gated to the respiratory cycle.

ONSD was scheduled at 10 perioperative time points: Preop, the awake preoperative baseline before induction; T0, after anaesthetic induction; T1, after initiation of CPB; T2, approximately 15 min after cooling was initiated during CPB; T3, at the deepest hypothermia; T4, after removal of the aortic cross-clamp; T5, during separation from CPB; T6, after sternal closure and before transfer to the ICU; Postop 1 h, within the first hour after ICU admission; and Post-extubation, after tracheal extubation. T6 was obtained after protamine reversal, decannulation, surgical haemostasis, and sternal closure. The interval between separation from CPB (T5) and T6 was not recorded as a study variable; neither was the exact interval from tracheal extubation to the post-extubation measurement.

Regional cerebral oxygen saturation was monitored bilaterally with an INVOS 5100C cerebral oximeter (Medtronic, Minneapolis, MN, USA) and adult forehead sensors. Near-infrared spectroscopy denotes the monitoring method, and rSO_2_ denotes the recorded variable. For analysis, the mean of the right and left sensors was used at eight points, from Preop to T6; the cerebral oximeter was not retained for the two postoperative ONSD time points.

All ONSD measurements were obtained by a single anaesthesiologist with 10 years of clinical experience, including point-of-care ocular sonography. The operator did not participate in delirium or MoCA assessments; both assessors were blinded to ONSD. The ONSD operator was not blinded to measurements already entered during the same operation. Measurements were recorded on the case-report form at acquisition. Ultrasound images and individual replicate measurements were not archived; consequently, eyeball transverse diameter (ETD) could not be measured retrospectively, the ONSD/ETD ratio could not be calculated, and intra-observer remeasurement was not possible.

### Delirium and cognitive assessment

Delirium was assessed with the CAM-ICU assessment (19) twice daily in the ICU and once daily on the ward, from ICU admission until postoperative day 10, hospital discharge, or death, whichever occurred first. The same instrument was retained on the ward to maintain consistency in the case definition throughout the observation window. Any altered mental status reported by the treating team between scheduled assessments prompted an additional CAM-ICU assessment. A participant was classified as having postoperative delirium after at least one positive CAM-ICU assessment during this period, with every positive assessment confirmed by psychiatry and/or neurology consultation. Participants with at least one valid CAM-ICU assessment and no positive result during their observable period were classified as not having documented delirium. Two participants never reached a CAM-ICU-assessable state before death; both also remained intubated throughout. Their delirium status was therefore classified as indeterminate rather than absent.

All scheduled CAM-ICU assessments were performed by one board-certified anaesthesiologist trained in the instrument and blinded to the ONSD data. Arousal was graded with the Richmond Agitation–Sedation Scale (RASS) (20) before each assessment. The CAM-ICU was administered only when RASS was −3 or higher; a patient at RASS −4 or −5 was recorded as unable to assess and reassessed at the next scheduled time. Delirium motor subtype was assigned by the psychiatrist or neurologist who confirmed the diagnosis at the first positive assessment. The onset date of delirium and the episode duration were not prospectively recorded as separate study variables.

The MoCA was administered and scored according to its standard instructions (21). One point was added for participants with 12 or fewer years of formal education, with the total score capped at 30. The preoperative and postoperative assessments were conducted by the same trained assessor, who was blinded to ONSD and did not perform the CAM-ICU assessments. The postoperative assessment was performed once the patient was lucid and free of active delirium, before discharge, and within the first 10 postoperative days. A fixed postoperative day was not pre-specified, and the exact assessment day was not recorded. MoCA was not administered during active delirium or when the patient could not participate validly; neither of the two patients with indeterminate delirium status reached a state permitting valid postoperative MoCA administration.

### Statistical analysis

The retained perioperative cohort comprised the 53 participants remaining after the two intraoperative exclusions. Outcome-independent analyses used all available observations from this cohort. Primary analyses requiring delirium status included the 51 participants with assessable delirium status (17 with and 34 without documented delirium); the two participants with indeterminate outcomes were excluded. No confirmatory multiplicity hierarchy had been pre-specified; all inferential analyses were therefore treated as exploratory. Missing observations were not imputed. Analyses requiring a specific measurement used the available cases at that time point, whereas linear mixed-effects models used all available repeated observations. MoCA change analyses were restricted to the same 51 participants, who had both a preoperative and a valid postoperative assessment. In the retained perioperative cohort, the serial ONSD dataset contained 527 of 530 scheduled bilateral time-point pairs: one complete T4 assessment was missed because the investigator was unavailable, and post-extubation ONSD was unavailable in the two participants who were not extubated. Because these two participants were never extubated, post-extubation core temperature was not applicable, and time to extubation was not observed; therefore, both variables were summarised among the 51 participants who were extubated. The rSO_2_ dataset contained 423 of 424 scheduled bilateral time-point pairs because the same T4 assessment was missed. The complete T4 study assessment included ONSD, rSO_2_, mean arterial pressure, and blood-gas variables; core temperature at T4 remained available in all 53 participants. The delirium-status mixed model contained all 510 ONSD observations from the 51 participants with assessable delirium status, and the corresponding rSO_2_ group comparisons contained 408 observations.

Distributional assumptions were assessed using the Shapiro–Wilk test. Continuous variables were summarised as mean ± standard deviation (SD) when approximately normally distributed and as median [interquartile range (IQR)] otherwise; categorical variables were reported as numbers and percentages. Between-group comparisons used Welch's independent-samples *t*-test or the Mann–Whitney *U*-test for continuous variables, depending on distribution, Fisher's exact test for 2 × 2 categorical tables, and the Fisher–Freeman–Halton exact test for larger contingency tables.

Descriptive summaries and time-point analyses used all available observations from the full cohort; the Friedman omnibus tests were restricted to participants with complete serial data (ONSD, *n* = 51; rSO_2_, *n* = 52). Overall ONSD and rSO_2_ trajectories were assessed with these complete-case Friedman tests. If an omnibus test was significant, each later time point was compared with the awake preoperative value using all available paired observations and the Wilcoxon signed-rank test. Holm–Bonferroni adjustment (22) was applied separately to the nine baseline-referenced ONSD comparisons and the seven baseline-referenced rSO_2_ comparisons, and the effect size was calculated as *r* = |z|/√*n*. For exploratory comparisons between the 34 patients without and the 17 with documented delirium, Mann–Whitney *U*-tests were used, and Holm adjustment was applied separately across the family of 10 ONSD comparisons and the family of eight rSO_2_ comparisons. Both raw and adjusted *p*-values were reported.

Serial ONSD in the 51 participants with assessable delirium status was analysed using a linear mixed-effects model with categorical time and delirium group as fixed effects and a random intercept for participant. The average group difference was estimated from the main-effects model by restricted maximum likelihood. A second model added the group-by-time interaction; the main-effects and interaction models were refitted by maximum likelihood and compared with a nine-degree-of-freedom likelihood ratio test. The interaction analysis was exploratory and was not used to designate a superior time point.

Global inter-eye agreement was assessed using the Bland–Altman method adapted for repeated measurements (23). The variance of the right-minus-left differences was partitioned into between-patient and within-patient components with a linear mixed model, and limits of agreement were derived from the total variance. The confidence interval for the mean difference was obtained from 10,000 cluster-bootstrap resamples of patients rather than individual observations (random seed 42). Proportional bias was assessed by regressing the inter-eye difference on the mean of the paired eye measurements with cluster-robust standard errors. To avoid treating repeated occasions within the same patient as independent targets, the intraclass correlation coefficient [ICC(A,1)]—a two-way random-effects, absolute-agreement, single-measurement coefficient—was calculated separately at each study time point (24). These coefficients were interpreted as inter-eye concordance rather than as intra- or inter-observer reliability.

Associations with delirium were estimated in the 51 evaluable participants using Firth penalised logistic regression (25); 95% confidence intervals (CIs) were obtained from the profile penalised likelihood, and *p*-values were obtained from penalised likelihood ratio tests. After review of univariable comparisons and clinical plausibility, a limited data-informed set was selected to represent ONSD, circulatory-arrest exposure, hypothermia, preoperative cognition, and alcohol use while excluding postoperative consequences of delirium and avoiding multiple closely related procedural variables. The exploratory five-covariate model therefore comprised T6 ONSD, TCA duration, nadir core temperature, preoperative MoCA, and alcohol use. TCA duration was coded as 0 min in the 27 evaluable patients who did not undergo TCA; its descriptive comparison within the 24 evaluable patients who underwent TCA was a separate estimand. Because the number of delirium events was small, a reduced model containing T6 ONSD and preoperative MoCA and a model containing T6 ONSD alone were also fitted. These models were not pre-specified and were not interpreted as establishing an independent predictor.

To distinguish baseline levels from intraoperative change, separate Firth models were evaluated: Preop ONSD, T0 ONSD, T6 ONSD, and change from baseline (T6 minus Preop). Baseline-adjusted models included T6 with Preop and T6 with T0; an algebraically equivalent parameterisation included Preop and T6 minus Preop. Supportive nested-model likelihood ratio tests and Akaike information criterion comparisons were obtained from ordinary maximum-likelihood logistic models; baseline-adjusted coefficient estimates were obtained separately with Firth penalised logistic regression. *Post-hoc* sensitivity analyses adjusted the T6 ONSD association separately for concurrent PaCO_2_, mean arterial pressure, haematocrit, pH, core temperature, nadir core temperature, TCA duration, TCA exposure, methylprednisolone exposure, and mannitol exposure; an additional model jointly included concurrent PaCO_2_, haematocrit, and mean arterial pressure. TCA-stratified estimates and an ONSD-by-TCA interaction were also examined. These analyses assessed robustness but could not exclude residual confounding.

Exploratory time-matched associations between ONSD and core temperature, PaCO_2_, mean arterial pressure, haematocrit, and pH were assessed in the full cohort with Spearman’s rank correlation coefficients using available pairs; Holm adjustment was applied separately within each physiological-variable family.

Because both indeterminate outcomes occurred in participants who died, missingness could be informative. In addition to the primary analysis of participants with assessable delirium status, each indeterminate outcome was independently assigned as absent or present, producing four deterministic scenarios (00, 01, 10, and 11). For each scenario, we repeated the serial ONSD main-effects and time-by-group mixed models, the univariable T6 Firth model, the receiver operating characteristic (ROC) analysis of T6, including its area under the curve (AUC), the performance of the fixed 4.90-mm operating point, and the apparent five-variable model AUC. These assignments were sensitivity analyses rather than observed outcomes and were used to assess the dependence of the principal conclusions on the two unobserved outcomes (Supplementary Table S11).

Discrimination was quantified by ROC curves and AUCs with 95% confidence intervals. Correlated AUCs for Preop, T0, T6, and change from Preop were compared using the DeLong method (26). Any operating point was selected using the Youden index in the same dataset and was therefore labelled as internally derived and exploratory. Sensitivity, specificity, positive predictive value, and negative predictive value were reported with Wilson confidence intervals (27). Internal validation of the penalised models used bootstrap resampling with Harrell optimism correction (28); 1,000 bootstrap resamples were requested; valid finite fits were retained, and the number of fits retained was reported for each model. The same fixed predictor set was refitted with Firth regression in each resample, and calibration was assessed using the calibration intercept, slope, and Brier score. A four-group Hosmer–Lemeshow statistic was reported only as a low-power supplementary calibration check. Initial univariable screening and selection of T6 from the candidate time points were not repeated in the bootstrap loop, so the correction was conditional on those data-informed choices and incomplete. The Youden operating-point analysis used 2,000 valid bootstrap resamples, with the threshold reselected within each resample; selection of T6 as the target time point was not repeated. Random seed 42 was fixed for both procedures. Externally validated performance was not estimated.

All tests were two-sided; *p* < 0.05 was used as the descriptive threshold within the exploratory framework. No additional multiplicity adjustment was applied across the exploratory regression, ROC, sensitivity, or subgroup model families. The original descriptive analyses were checked against the locked dataset maintained in IBM SPSS Statistics version 26.0. Reproducible revision analyses were performed in Python 3.12.13 using NumPy 1.26.4, pandas 2.3.3, SciPy 1.17.1, statsmodels 0.14.6, pyreadstat 1.3.5, scikit-learn 1.7.2, firthlogist 0.5.0, and pingouin 0.5.5. Random seed 42 was used for all resampling procedures. Generative AI tools were used for language editing and to assist with drafting statistical analysis code, as disclosed in the Generative AI statement; all analyses were rerun using the locked dataset, and the numerical outputs were reviewed by the authors before reporting.

## Results

### Study population

Sixty participants were assessed for eligibility. Five were excluded before enrolment [the study investigator was unavailable in two cases, one participant declined participation, and preoperative Montreal Cognitive Assessment (MoCA) testing could not be completed in two cases]. Fifty-five participants were enrolled and underwent surgery. Two enrolled participants were excluded intraoperatively because separation from cardiopulmonary bypass could not be achieved, leaving 53 participants in the full study cohort (Figure 1). Delirium status was assessable in 51 participants: 17 had documented postoperative delirium (33.3%), and 34 had no documented delirium during their observable period. Two participants never reached a CAM-ICU-assessable state before death and were classified as having indeterminate delirium status; both also remained intubated throughout. Clinical subtype classification recorded 13 hypoactive (76.5%), three hyperactive (17.6%), and one mixed (5.9%) cases among the 17 participants with documented delirium.

**Figure 1:**
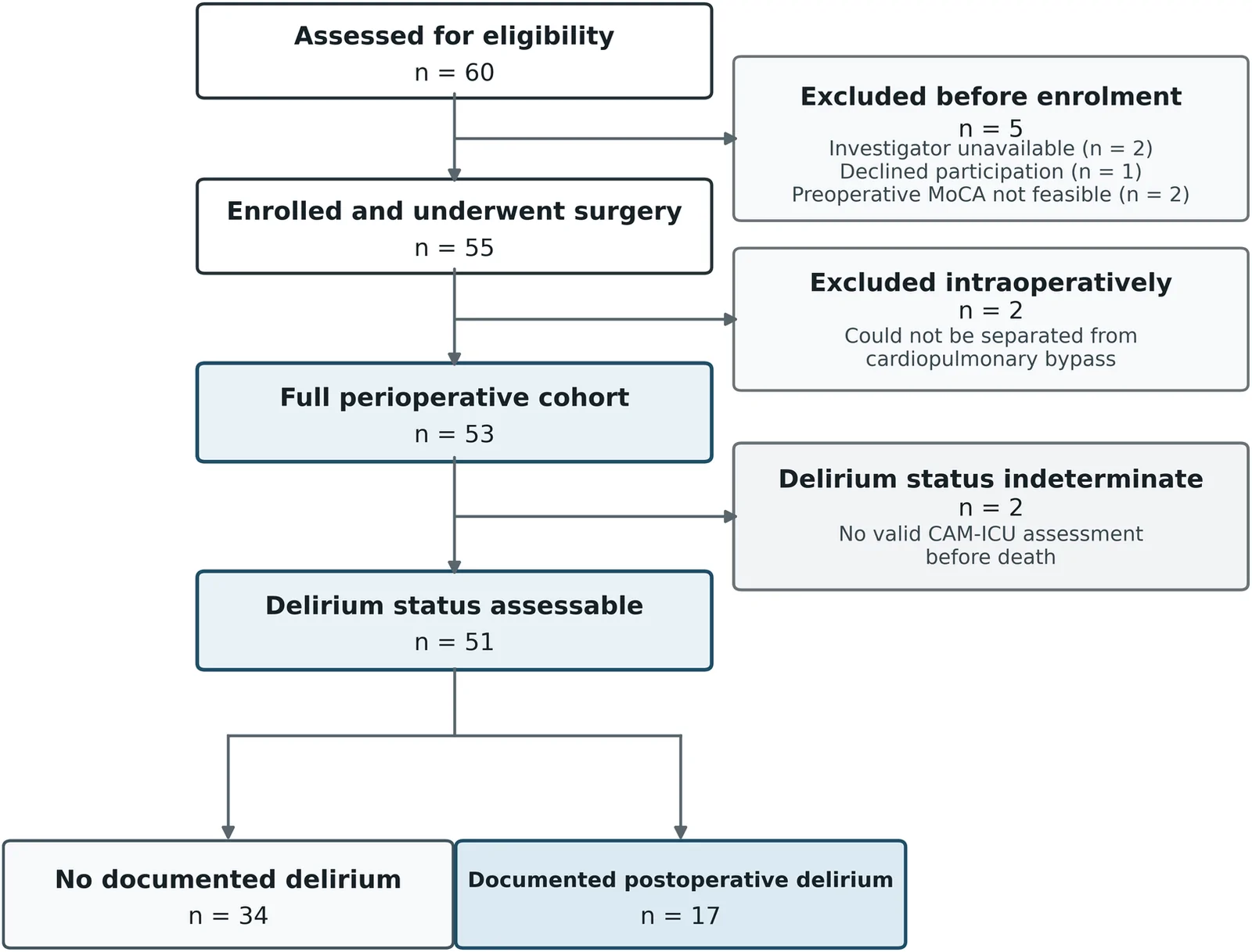
Participant flow. The full perioperative cohort comprised 53 participants. Delirium-status analyses included 51 assessable participants; two participants who never had a valid CAM-ICU assessment before death were classified as indeterminate.

Participant and perioperative characteristics among the 51 participants with assessable delirium status are summarised in Table 1. Preoperative MoCA scores were 22.8 ± 2.3 in participants without documented delirium and 21.5 ± 1.7 in those with documented delirium (*p* = 0.025). Total circulatory arrest was performed in 13/34 (38.2%) and 11/17 (64.7%) participants (*p* = 0.136). Among the 24 participants with assessable delirium status who underwent circulatory arrest, the duration was 11.0 [9.5–15.0] min without documented delirium and 13.0 [11.0–18.5] min with documented delirium (*p* = 0.172). Expanded characteristics and available-case NRS and analgesia data are provided in Supplementary Tables S1 and S8.

**Table 1:** Selected cohort characteristics by delirium status.

Variable	All participants (*n* = 53)	No delirium (*n* = 34)	Delirium (*n* = 17)	*p*-Value
Age (years)	58.6 ± 9.6	57.3 ± 9.2	59.8 ± 10.0	0.382
Female sex, *n* (%)	18 (34%)	14 (41%)	4 (24%)	0.352
Body mass index (kg/m^2^)	27.6 ± 2.9	27.5 ± 3.2	27.9 ± 2.5	0.643
Ejection fraction (%)	55.0 [50.0–60.0]	60.0 [50.0–60.0]	55.0 [50.0–60.0]	0.279
EuroSCORE II	5.0 [3.0–5.0]	4.0 [3.0–5.0]	5.0 [3.0–5.0]	0.503
Diabetes mellitus, *n* (%)	7 (13%)	3 (9%)	3 (18%)	0.387
Smoking, *n* (%)	27 (51%)	16 (47%)	10 (59%)	0.555
Alcohol use, *n* (%)	4 (8%)	1 (3%)	3 (18%)	0.102
Preoperative MoCA (points)	22.3 ± 2.4	22.8 ± 2.3	21.5 ± 1.7	0.025
Cardiopulmonary bypass time (min)	134.0 [112.0–160.0]	118.0 [102.2–159.2]	143.0 [124.0–160.0]	0.204
Aortic cross-clamp time (min)	95.6 ± 39.0	90.5 ± 38.8	101.5 ± 37.7	0.338
Operative time (min)	306.5 ± 72.3	293.4 ± 73.1	323.2 ± 67.4	0.157
Total circulatory arrest, *n* (%)	26 (49%)	13 (38%)	11 (65%)	0.136
Duration of circulatory arrest (min), TCA patients	11.5 [10.0–16.8]	11.0 [9.5–15.0]	13.0 [11.0–18.5]	0.172
Nadir core temperature (°C)	30.0 [28.0–32.0]	31.0 [28.0–32.0]	28.0 [26.0–30.0]	0.081
Time to extubation (min)	370.0 [340.0–420.0]	387.5 [351.2–445.0]	345.0 [340.0–385.0]	0.062
Intensive care unit stay (days)	2.0 [2.0–3.0]	2.0 [2.0–3.0]	3.0 [2.0–3.0]	0.280
Time to discharge or in-hospital death (days)	7.0 [7.0–8.0]	8.0 [7.0–8.0]	7.0 [7.0–8.0]	0.169

The overall column includes all 53 participants; group summaries and *p*-values include 51 participants with assessable delirium status. Values are mean ± SD, median [IQR], or *n* (%). Circulatory-arrest duration applies to 26 participants overall and 24 in group comparisons (13 without and 11 with delirium). Time to extubation is available for 51 because two participants were never extubated. *p*-Values are unadjusted.

Neither patient with indeterminate delirium status reached a state permitting valid postoperative MoCA administration. Post-extubation ONSD and core temperature were not obtained in either patient. In one of these patients, T4 ONSD, rSO_2_, mean arterial pressure, and blood gas measurements were unavailable because the investigator was not present at the time. Consequently, the full-cohort datasets contained 527 of 530 scheduled bilateral ONSD time-point pairs and 423 of 424 scheduled bilateral rSO_2_ time-point pairs. The primary delirium-status analyses contained 510 ONSD and 408 rSO_2_ observations from the 51 participants with assessable delirium status. No missing values were imputed.

### Perioperative ONSD course and delirium

In the full-cohort serial dataset, the complete-case Friedman analysis among the 51 participants with measurements at all 10 points showed a change in ONSD over time (*χ*^2^ = 47.36, df = 9, *p* < 0.001; Table 2A). The cohort median was 4.30 mm [4.00–4.70] before surgery and 4.30 mm [4.00–4.70] at T0. After Holm correction, ONSD was higher than the awake preoperative value at T1–T6, 1 h postoperatively, and after extubation (adjusted *p* ≤ 0.023); T0 did not differ from the preoperative value (adjusted *p* = 0.592). In the primary main-effects mixed model of the 510 observations from the 51 participants with assessable delirium status, average ONSD across the perioperative course was 0.250 mm higher in participants with documented delirium (95% CI 0.031–0.470; *p* = 0.025). Adding the time-by-group interaction improved model fit in this analysis (likelihood ratio *χ*^2^ = 22.37, df = 9; *p* = 0.008; Figure 2).

**Table 2:** Serial bilateral ONSD and rSO_2_ in the cohort.

Time point	*n*	Value, median [IQR] (mm or %)	Median change from preop (mm or percentage points)	Raw *p*	Holm *p*	Effect *r*
Panel A: ONSD (mm)						
Preop	53	4.30 [4.00–4.70]	–	–	–	–
T0	53	4.30 [4.00–4.70]	+0.00	0.592	0.592	0.07
T1	53	4.40 [4.10–4.80]	+0.10	<0.001	0.003	0.48
T2	53	4.45 [4.15–5.00]	+0.15	<0.001	0.004	0.48
T3	53	4.60 [4.10–5.00]	+0.20	0.008	0.023	0.37
T4	52	4.55 [4.25–4.81]	+0.30	<0.001	0.003	0.49
T5	53	4.55 [4.30–4.90]	+0.20	<0.001	0.004	0.46
T6	53	4.60 [4.30–4.95]	+0.30	<0.001	0.004	0.47
Postop 1 h	53	4.50 [4.30–4.90]	+0.15	0.001	0.006	0.44
Post-extubation	51	4.60 [4.30–4.82]	+0.15	0.008	0.023	0.37
Panel B: rSO_2_ (%)						
Preop	53	66.0 [60.5–71.5]	–	–	–	–
T0	53	66.0 [60.5–71.5]	+0.0	0.755	0.755	0.04
T1	53	67.5 [61.0–75.5]	+1.0	0.201	0.401	0.18
T2	53	63.0 [56.0–69.0]	−4.0	0.004	0.019	0.40
T3	53	60.0 [52.0–66.5]	−5.5	<0.001	<0.001	0.63
T4	52	64.0 [55.9–66.0]	−4.8	0.003	0.018	0.41
T5	53	65.0 [57.0–72.0]	−2.5	0.087	0.347	0.24
T6	53	66.0 [56.5–70.5]	−1.0	0.132	0.396	0.21

Panel A (ONSD): ONSD is the bilateral mean (mm). Available observations were used; Friedman included 51 complete cases. Preop comparisons used Wilcoxon signed-rank tests with Holm correction across nine tests; *r* = |*z*|/√*n*. Friedman *χ*^2^ = 47.36, df = 9, *p* < 0.001. Panel B (rSO_2_): rSO_2_ is the bilateral mean (%); changes are percentage points. Available observations were used; Friedman included 52 complete cases. Preop comparisons used Wilcoxon signed-rank tests with Holm correction across seven tests; *r* = |*z*|/√*n*. Friedman *χ*^2^ = 51.94, df = 7, *p* < 0.001.

**Figure 2:**
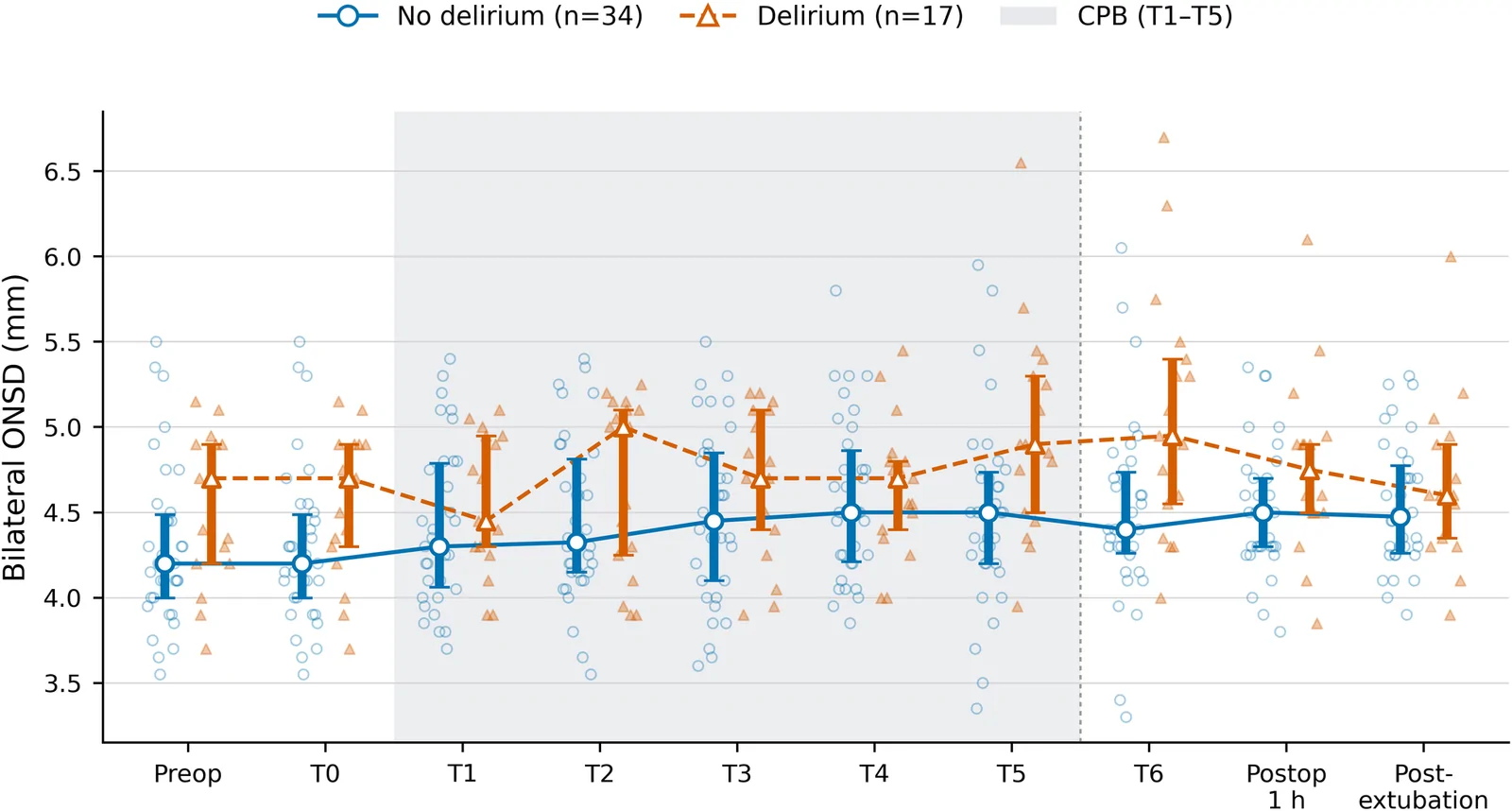
Perioperative bilateral ONSD by delirium status. Faint, horizontally jittered symbols show individual bilateral ONSD observations among 51 participants with assessable delirium status; open circles with a solid line denote no documented delirium (*n* = 34), and triangles with a dashed line denote documented delirium (*n* = 17). Large symbols show medians, and vertical bars show the interquartile range; lines join group medians. Grey shading marks cardiopulmonary bypass (T1–T5). T6 was measured after sternal closure and before transfer to the intensive care unit.

At individual time points, the smallest unadjusted between-group *p*-values occurred at T5 (*p* = 0.008), T6 (*p* = 0.009), and T0 (*p* = 0.030). None remained significant after Holm correction across the family of 10 comparisons (adjusted *p* = 0.078, 0.079, and 0.239, respectively; Table 3A). The unadjusted between-group comparisons yielded *p* = 0.147 for T6-minus-Preop change and *p* = 0.549 for peak intraoperative ONSD minus Preop (Table 3B).

**Table 3:** Serial bilateral ONSD and change measures by delirium status.

Time point or measure	No delirium, median [IQR] (mm)	Delirium, median [IQR] (mm)	Raw *p*	Holm *p*/multiplicity
Panel A: serial ONSD (mm)				
Preop	4.20 [4.00–4.49] (*n* = 34)	4.70 [4.20–4.90] (*n* = 17)	0.077	0.460
T0	4.20 [4.00–4.49] (*n* = 34)	4.70 [4.30–4.90] (*n* = 17)	0.030	0.239
T1	4.30 [4.06–4.79] (*n* = 34)	4.45 [4.30–4.95] (*n* = 17)	0.234	1.000
T2	4.33 [4.15–4.81] (*n* = 34)	5.00 [4.25–5.10] (*n* = 17)	0.230	1.000
T3	4.45 [4.10–4.85] (*n* = 34)	4.70 [4.40–5.10] (*n* = 17)	0.208	1.000
T4	4.50 [4.21–4.86] (*n* = 34)	4.70 [4.40–4.80] (*n* = 17)	0.384	1.000
T5	4.50 [4.20–4.74] (*n* = 34)	4.90 [4.50–5.30] (*n* = 17)	0.008	0.078
T6	4.40 [4.26–4.74] (*n* = 34)	4.95 [4.55–5.40] (*n* = 17)	0.009	0.079
Postop 1 h	4.50 [4.30–4.70] (*n* = 34)	4.75 [4.50–4.90] (*n* = 17)	0.059	0.414
Post-extubation	4.47 [4.26–4.78] (*n* = 34)	4.60 [4.35–4.90] (*n* = 17)	0.389	1.000
Panel B: change in ONSD (mm)				
Postop 1 h−Preop	+0.15 [−0.04–0.49] (*n* = 34)	+0.10 [0.00–0.55] (*n* = 17)	0.968	Exploratory and unadjusted; outside 10-time-point family
Post-extubation−Preop	+0.15 [0.01–0.52] (*n* = 34)	+0.05 [−0.20–0.40] (*n* = 17)	0.285	Exploratory and unadjusted; outside 10-time-point family
T6−Preop	+0.28 [−0.05–0.45] (*n* = 34)	+0.60 [−0.10–0.80] (*n* = 17)	0.147	Exploratory and unadjusted; outside 10-time-point family
Intraoperative peak−Preop	+0.45 [0.21–0.74] (*n* = 34)	+0.60 [0.25–0.80] (*n* = 17)	0.549	Exploratory and unadjusted; outside 10-time-point family

The 51 participants with assessable delirium status contributed to these comparisons. ONSD values and changes are reported (in mm) as median [IQR]. Time-point comparisons used Mann–Whitney *U*-tests with Holm adjustment across 10 comparisons; change comparisons were exploratory and unadjusted. T6 was measured after sternal closure.

Across the four deterministic assignments of the two indeterminate outcomes, the average mixed-model group difference ranged from 0.237 to 0.248 mm (*p*-values ranged from 0.020 to 0.029), the univariable T6 odds ratio ranged from 2.86 to 4.23 (*p*-values ranged from 0.002 to 0.016), and the T6 AUC ranged from 0.692 to 0.743. At the fixed 4.90-mm operating point, sensitivity ranged from 0.556 to 0.611 and specificity from 0.800 to 0.829. In contrast, the time-by-group interaction *p*-values ranged from <0.001 to 0.094, indicating that the average association and operating-point performance were similar across assignments but the evidence that the temporal pattern differed by group was assignment-sensitive (Supplementary Table S11).

### Baseline, T6, and change-from-baseline analyses

In univariable Firth models, the odds ratios (ORs) for delirium per 1-mm higher ONSD were 2.64 (95% CI 0.79–9.68; *p* = 0.114) for Preop, 3.26 (0.95–12.87; *p* = 0.061) for T0, and 3.75 (1.44–12.32; *p* = 0.005) for T6. The OR per 1-mm greater T6-minus-Preop change was 2.35 (0.92–7.21; *p* = 0.075; Table 4C). The corresponding AUCs were 0.654 (95% CI 0.492–0.816), 0.689 (0.528–0.849), 0.728 (0.574–0.881), and 0.626 (0.441–0.811) (Figure 3). Paired DeLong comparisons provided no clear evidence that T6 discrimination differed from Preop (*p* = 0.399), T0 (*p* = 0.641), or change from Preop (*p* = 0.209).

**Table 4:** Firth penalised logistic regression models for postoperative delirium.

Model	Term	*n*	Events	OR	95% CI	*p*-Value
Panel A: five-variable model						
Five-variable model	T6 ONSD per mm	51	17	3.73	1.32–15.72	0.010
Five-variable model	TCA duration per min	51	17	1.04	0.95–1.15	0.364
Five-variable model	Nadir core temperature per °C	51	17	0.86	0.62–1.18	0.285
Five-variable model	Preoperative MoCA per point	51	17	0.72	0.48–1.00	0.052
Five-variable model	Alcohol use: yes vs. no	51	17	3.77	0.35–65.52	0.272
Panel B: reduced models						
Reduced: T6 ONSD + preoperative MoCA	T6 ONSD per mm	51	17	3.50	1.38–11.14	0.007
Reduced: T6 ONSD + preoperative MoCA	Preoperative MoCA per point	51	17	0.74	0.51–1.00	0.052
T6 ONSD alone	T6 ONSD per mm	51	17	3.75	1.44–12.32	0.005
Panel C: baseline and change models						
Preoperative ONSD alone	Preoperative ONSD per mm	51	17	2.64	0.79–9.68	0.114
T0 ONSD alone	T0 ONSD per mm	51	17	3.26	0.95–12.87	0.061
T6 ONSD alone (comparison)	T6 ONSD per mm	51	17	3.75	1.44–12.32	0.005
T6 minus Preop alone	T6 minus Preop per mm	51	17	2.35	0.92–7.21	0.075
T6 adjusted for Preop	T6 ONSD per mm	51	17	3.29	1.19–11.90	0.020
T6 adjusted for Preop	Preoperative ONSD per mm	51	17	1.35	0.31–5.68	0.676
T6 adjusted for T0	T6 ONSD per mm	51	17	3.13	1.09–11.42	0.033
T6 adjusted for T0	T0 ONSD per mm	51	17	1.55	0.34–7.05	0.562
Re-parameterised Preop + change	Preoperative ONSD per mm	51	17	4.45	1.16–20.28	0.029
Re-parameterised Preop + change	T6 minus Preop per mm	51	17	3.29	1.19–11.90	0.020

ORs are per stated unit; 95% CIs are profile penalised likelihood intervals, and *p*-values are from penalised likelihood ratio tests. Each model includes 51 participants with assessable delirium status and 17 events.

CI, confidence interval; MoCA, Montreal Cognitive Assessment; ONSD, optic nerve sheath diameter; OR, odds ratio; TCA, total circulatory arrest.

**Figure 3:**
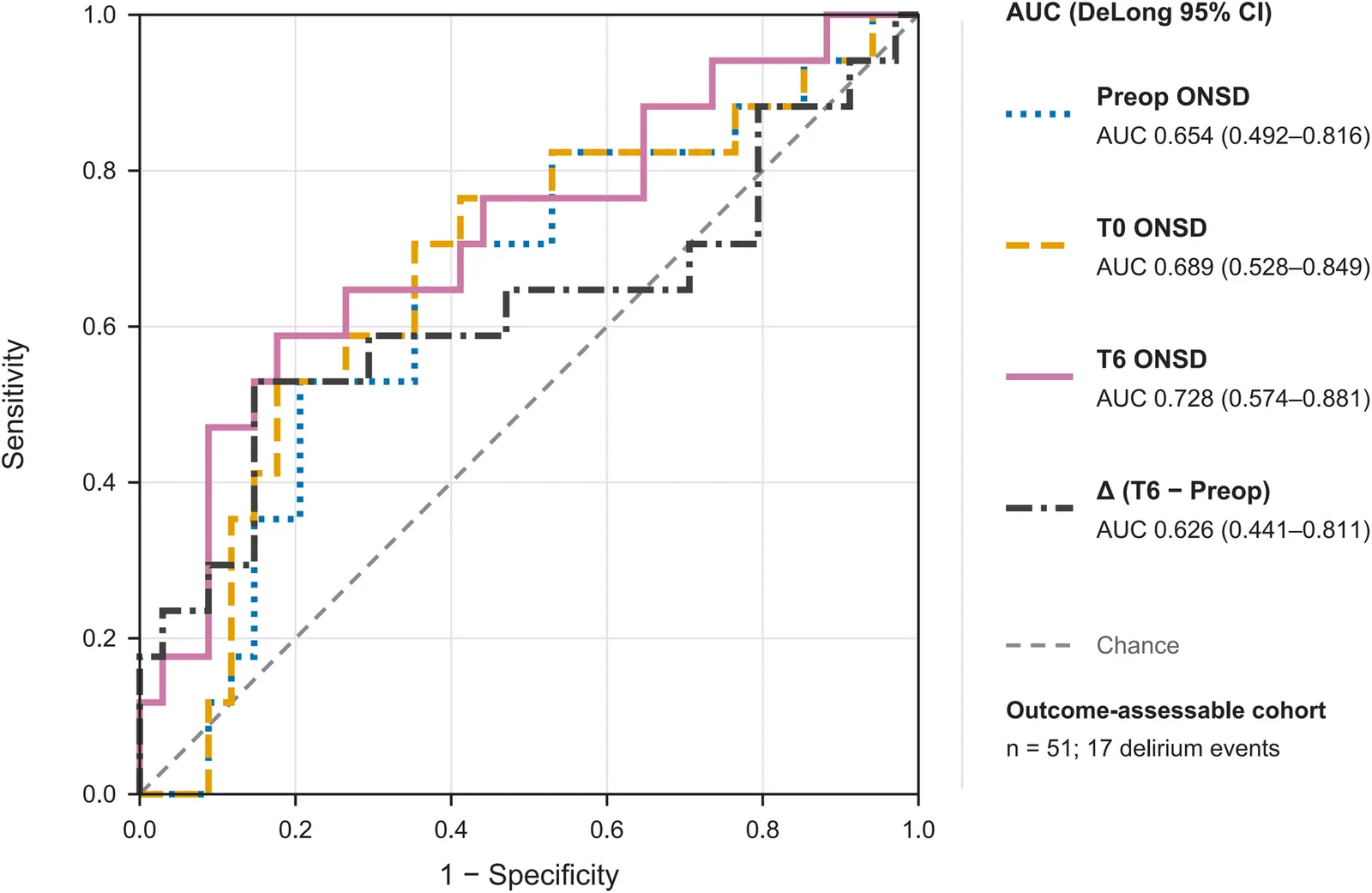
ROC curves for postoperative delirium. Preop, T0, T6, and T6-minus-Preop ONSD in 51 participants with assessable delirium status (17 events); the 95% CIs shown in the legend were calculated using the DeLong method.

When Preop and T6 ONSD were entered together, the T6 estimate was 3.29 per mm (95% CI 1.19–11.90; *p* = 0.020) and the Preop estimate was 1.35 (0.31–5.68; *p* = 0.676). In the corresponding T0-adjusted model, the T6 estimate was 3.13 (1.09–11.42; *p* = 0.033) and the T0 estimate was 1.55 (0.34–7.05; *p* = 0.562). Re-expression of the Preop-adjusted model as Preop plus T6-minus-Preop change yielded estimates of 4.45 (1.16–20.28; *p* = 0.029) for Preop and 3.29 (1.19–11.90; *p* = 0.020) for change (Table 4C). This algebraically equivalent parameterisation does not demonstrate that an intraoperative increase caused delirium. In exploratory nested comparisons of ordinary logistic regression models, adding T6 improved fit beyond Preop (likelihood ratio *p* = 0.015) and T0 (*p* = 0.025), whereas adding Preop to T6 did not (*p* = 0.711; Supplementary Table S3). All estimates were imprecise, and these analyses did not establish superiority of any measurement time point.

### Exploratory penalised models and discrimination

The data-informed five-variable Firth model included T6 ONSD, circulatory-arrest duration (coded as 0 min in the 27 evaluable patients without circulatory arrest), nadir core temperature, preoperative MoCA, and alcohol use (Table 4A). With 17 events for five covariates, T6 ONSD had an odds ratio of 3.73 per mm (95% CI 1.32–15.72; *p* = 0.010); the estimates for the other covariates were imprecise, and their confidence intervals included the null. In a reduced model containing T6 ONSD and preoperative MoCA, the respective odds ratios were 3.50 (1.38–11.14; *p* = 0.007) and 0.74 per MoCA point (0.51–1.00; *p* = 0.052; Table 4B). The five-variable model had an apparent AUC of 0.839 and a conditional bootstrap optimism-corrected AUC of 0.778. Of 1,000 requested bootstrap samples, 984 yielded finite fits; 27 of these produced a convergence warning but finite estimates and were retained, whereas 16 failed. The apparent calibration intercept and slope were 0.089 and 1.234, respectively, and the optimism-corrected slope was 0.814; the apparent Brier score was 0.146. The four-group Hosmer–Lemeshow statistic was 1.53 (df = 2; *p* = 0.464), but this test had low power in the small sample. The bootstrap refitted a fixed predictor set and did not repeat the preceding variable and time-point selection; the correction therefore remains incomplete (Supplementary Table S2A and Supplementary Figure S2).

The sample-derived T6 operating point was 4.90 mm. Its apparent sensitivity was 0.59 (95% CI 0.36–0.78) and specificity was 0.82 (0.66–0.92). When operating-point selection was repeated in 2,000 bootstrap samples, the median selected value was 4.90 mm (interquartile range 4.75–5.10; 2.5th–97.5th percentiles 4.30–5.30), and optimism-corrected sensitivity and specificity were 0.52 and 0.80 (Supplementary Table S2B). The bootstrap did not repeat the selection of T6 from among the study time points. This operating point was derived and evaluated in the same 51 participants with assessable delirium status and is reported only to facilitate replication; it is not a threshold for intracranial hypertension and is not suitable for individual risk classification without external validation.

### Cerebral oximetry and physiological analyses

In the full-cohort serial dataset, the complete-case Friedman analysis among the 52 participants with measurements at all eight points showed a change in mean bilateral rSO_2_ over time (*χ*^2^ = 51.94, df = 7, *p* < 0.001; Table 2B). Relative to Preop, rSO_2_ was lower at T2, T3, and T4 after Holm correction (adjusted *p* = 0.019, *p* < 0.001, and *p* = 0.018, respectively). Among the 51 participants with assessable delirium status, the smallest unadjusted between-group *p*-value occurred at T2 (*p* = 0.025), but it did not remain significant after Holm correction across eight between-group comparisons (adjusted *p* = 0.201). No other point-specific rSO_2_ comparison survived correction (Supplementary Table S9).

Across T0–T6 in the full cohort, the mean PaCO_2_ ranged from 40.6 to 45.8 mmHg, the mean arterial pressure ranged from 65.4 to 102.0 mmHg, the haematocrit ranged from 28.1% to 44.3%, and the pH from 7.33 to 7.41; the mean core temperature across the 10 study time points ranged from 29.2 °C to 36.8 °C (Supplementary Table S6A). Point-specific central venous pressure values were unavailable. In separate Firth sensitivity models among the 51 participants with assessable delirium status, adjustment for concurrent PaCO_2_, mean arterial pressure, haematocrit, pH, or core temperature; nadir core temperature; circulatory-arrest duration or exposure; and methylprednisolone or mannitol exposure yielded T6 odds-ratio estimates from 3.52 to 4.21 per mm (all *p* = 0.005–0.008; Supplementary Table S4). A joint model including concurrent PaCO_2_, haematocrit, and mean arterial pressure yielded an odds ratio of 3.87 (95% CI 1.43–13.40; *p* = 0.006). These sparse models do not exclude residual confounding.

All 26 participants in the full cohort who underwent total circulatory arrest received methylprednisolone, mannitol, and an additional thiopental bolus as part of the arrest protocol. Methylprednisolone and mannitol were also recorded in 19/27 and 16/27 participants without circulatory arrest, respectively; the recorded mannitol volumes are summarised separately (Supplementary Tables S5A–C). Among the 51 participants with assessable delirium status, the T6 ONSD odds ratio was 3.70 (95% CI 1.11–22.62; *p* = 0.032) in the 24-participant circulatory-arrest stratum and 3.12 (0.70–18.48; *p* = 0.139) in the 27-participant no-arrest stratum; there was no clear evidence of an ONSD-by-arrest interaction (interaction OR 1.19, 95% CI 0.14–11.75; *p* = 0.839). No full-cohort ONSD correlation with temperature, PaCO_2_, mean arterial pressure, haematocrit, or pH remained significant after within-family Holm correction (Supplementary Table S6B).

### Inter-eye ONSD concordance

Time-specific absolute-agreement ICC(A,1) estimates ranged from 0.58 at T4 to 0.88 at T6 (Supplementary Table S7). In the global repeated-measures Bland–Altman analysis of 527 paired right–left observations, the mean right-minus-left difference was −0.009 mm (95% patient-level cluster-bootstrap CI −0.073 to 0.050), and the limits of agreement were −0.727 to 0.708 mm. Twenty-seven observations (5.1%) fell outside these limits. There was no evidence of proportional bias after accounting for clustering by patient (slope 0.040; *p* = 0.490; Supplementary Figure S1). These analyses quantify inter-eye concordance and do not measure intra-observer reliability.

### Early postoperative MoCA change

Paired preoperative and postoperative MoCA measurements were available in 51 patients. The mean scores were 22.4 ± 2.2 before surgery and 22.4 ± 2.2 postoperatively (Wilcoxon signed-rank test, *p* = 0.959). The between-group comparison of change yielded *p* = 0.119. Eight of 51 patients (15.7%) met the study-defined exploratory threshold of a decrease of at least two points, with no evidence of a difference by delirium status (Fisher's exact test, *p* = 0.703; Supplementary Table S10). This threshold was not interpreted as a diagnosis of delayed neurocognitive recovery or postoperative neurocognitive disorder.

### Mortality

Ten-day mortality in the full cohort was 7/53 (13.2%). Five patients had documented delirium before death; the other two were never assessable with CAM-ICU, so their delirium status was indeterminate. Because there were only seven deaths, mortality is reported descriptively, and no inferential mortality analysis was undertaken (Supplementary Table S1).

## Discussion

In this exploratory prospective cohort of participants undergoing elective Bentall surgery, ONSD was higher on average across the perioperative course among the 17 participants with documented postoperative delirium than among the 34 participants without documented delirium. None of the 10 individual time-point comparisons remained significant after correction for multiplicity, and the exploratory unadjusted comparisons of T6-minus-Preop and peak-minus-Preop change provided no clear evidence of between-group differences. T6 ONSD was associated with delirium in the univariable Firth model, whereas the Preop and change estimates were imprecise; paired comparisons provided no clear evidence that the AUCs for Preop, T0, T6, and change differed. Assigning the two participants whose delirium status was indeterminate to each of the four possible outcome combinations produced similar average ONSD group effects and T6 associations, but the time-by-group interaction was sensitive to those assignments. Together, these observations support an association between ONSD magnitude and documented delirium status while leaving both its temporal pattern and the relative contributions of baseline anatomy or vulnerability and intraoperative change unresolved.

This distinction is important clinically and mechanistically. A preoperative measurement would be more actionable than a late intraoperative measurement if it proved reproducible in an external cohort. In the present data, however, the confidence intervals for baseline, post-induction, and T6 discrimination overlap substantially, and the study was not designed or powered to establish that any single time point was superior. The 95% confidence interval for the T6 estimate excluded the null value of 1 after separate adjustment for either Preop or T0 ONSD, whereas the corresponding Preop and T0 estimates were imprecise and included one. In the algebraically equivalent Preop-plus-change parameterisation, both terms were associated with delirium, but their wide confidence intervals reflected the limited information in 17 events. These data-informed analyses provide only sample-dependent evidence that T6 may contain information beyond the earliest measurements; they do not separate baseline anatomical susceptibility from perioperative physiology or establish an intraoperative mechanism. The results should therefore not be interpreted as evidence that delirium arose from a larger intraoperative increase in ONSD in the affected group.

The direction of the observed association is broadly compatible with prior perioperative studies. Serial ONSD changes have been described during extracorporeal circulation and under different perfusion strategies (12, 13), and Kaynar and colleagues reported an association between ONSD and delirium after open-heart surgery (14). Differences in surgical population, ultrasound acquisition, measurement timing, and outcome ascertainment limit direct numerical comparison. Our study extends this literature to elective Bentall surgery and includes bilateral serial measurements, but it does not validate a clinical prediction rule. T6 ONSD had only moderate apparent discrimination, and the sample-derived 4.90-mm operating point was selected and evaluated in the same 51 participants with assessable delirium status. The bootstrap distribution of the operating point was broad (2.5th–97.5th percentiles, 4.30–5.30 mm), and correcting its sensitivity and specificity for optimism reduced them from 0.59 to 0.52 and from 0.82 to 0.80, respectively. Because the bootstrap did not repeat the preceding selection of T6 from among the study time points, even these corrected values may remain optimistic. The operating point should not be used for individual risk classification without external validation.

Nor should 4.90 mm be interpreted as a threshold for intracranial hypertension. Ultrasound-derived ONSD is associated with raised intracranial pressure in selected neurocritical care populations, but it is an indirect, technique-dependent surrogate. Reported ONSD operating thresholds vary by population and acquisition technique and cannot be transferred directly to this cohort (5–7). Invasive intracranial pressure, neuroimaging, and biomarkers of cerebral oedema or blood–brain barrier injury were not obtained. Explanations involving intracranial compliance, cerebral oedema, or impaired autoregulation are therefore hypotheses rather than findings of this study.

The cerebral oximetry findings were also exploratory. rSO_2_ changed during surgery, but no point-specific difference between delirium groups survived correction across the eight comparisons. Near-infrared spectroscopy is influenced by extracranial signal contamination and does not provide a pure measure of cerebral oxygen delivery or extraction (29). The T6 ONSD estimate changed slightly in separate models adjusted for concurrent PaCO_2_, haematocrit, mean arterial pressure, pH, or core temperature, and in models accounting for circulatory arrest, nadir temperature, methylprednisolone, or mannitol. Estimates within the arrest and no-arrest strata were directionally similar but highly imprecise, and there was no clear evidence of an ONSD-by-arrest interaction. Temperature, PaCO_2_, haematocrit, mean arterial pressure, circulatory arrest management, and exposure to methylprednisolone, mannitol, thiopental, and glyceryl trinitrate could all influence the measurements. Adjustment for recorded variables in a cohort with only 17 events cannot eliminate residual confounding, particularly because drug administration and operative management were not randomised.

There was no clear evidence of a difference in early postoperative MoCA change by delirium status among the 51 patients with both assessments. This finding should be interpreted narrowly. A single MoCA administered on a variable postoperative day—and, for patients with delirium, only after resolution—serves as a screening measure rather than a comprehensive neuropsychological assessment or a diagnosis of delayed neurocognitive recovery or postoperative neurocognitive disorder (2, 21). The absence of a fixed assessment day, practice effects, and the unavailability of postoperative scores in two patients who never reached a state permitting valid MoCA administration further limit interpretation. The data therefore do not establish either the presence or the absence of longer-term cognitive consequences.

Several measurement limitations are central. Transbulbar B-mode ultrasound measures the outer optic nerve sheath complex but cannot reliably distinguish compressible distension of the perineural subarachnoid space from non-compressible solid enlargement; standardised A-scan with the 30° test is used in ophthalmic echography for this distinction (30, 31). A dedicated ophthalmic echography unit was unavailable, and the voluntary gaze deviation required for the 30° test was infeasible at the intraoperative time points under general anaesthesia and neuromuscular blockade. The closed-eyelid transpalpebral approach has also been criticised as a potential source of boundary-identification error (32, 33). The use of the small-parts preset rather than an ophthalmic preset was an additional protocol limitation. Images were not archived, so the eyeball transverse diameter could not be measured and the ONSD/ETD ratio could not be calculated (8, 9). Blinded remeasurement, intra-observer reliability, and minimal detectable change could not be estimated. Because intra-observer measurement error was not quantified, the clinical meaning of group differences measured in tenths of a millimetre remains uncertain. Time-specific inter-eye ICCs quantify right–left concordance rather than observer reliability. The repeated-measures limits of agreement were wide (−0.727–0.708 mm); these limits combine biological right–left differences with measurement disagreement and cannot substitute for an intra-observer error estimate.

Underlying aortic disease may introduce additional anatomical confounding. No patient had a documented diagnosis of Marfan syndrome or another heritable thoracic aortic disease, but systematic Ghent phenotyping, genetic testing, and detailed aetiological subclassification were not performed. Dural ectasia contributes to the systemic score in the revised Ghent nosology, and Marfan syndrome may alter ocular biometry (17, 34); a direct effect on ONSD independent of intracranial pressure has not been established. Undiagnosed heritable disease and variation in ocular or orbital anatomy therefore remain possible sources of residual confounding.

Other limitations include the single-centre design, the absence of an *a priori* sample-size calculation, only 17 documented delirium events, and the data-informed selection of the principal time point and multivariable covariates. Because the timing of delirium onset was not recorded separately, the Postop 1 h and post-extubation ONSD measurements may have followed the onset of delirium in some participants; the serial association therefore does not establish temporal precedence. Two enrolled patients who could not be separated from CPB were excluded because the serial protocol could not be completed; this post-enrolment attrition may limit generalisability to the most unstable patients. Firth penalisation reduces small-sample bias but cannot create information absent from the sample. Conditional bootstrap validation reduced the apparent discrimination of the five-variable model; because it held the selected variables and T6 time point fixed, it did not account for all sources of model-selection optimism. Delirium was assessed using CAM-ICU by one trained assessor until postoperative day 10, hospital discharge, or death. Two patients were never assessable using CAM-ICU before death and therefore had indeterminate delirium status; their exclusion from the primary outcome-dependent analyses could introduce bias from informative missingness. Across all four deterministic assignments of these two outcomes, the average ONSD group difference and fixed operating-point performance remained similar and the T6 association remained directionally stable, but the interaction *p* values ranged from <0.001 to 0.094 (Supplementary Table S11). This sensitivity analysis cannot recover the unobserved outcomes and indicates that the claim of differential ONSD trajectories is less robust than the overall association. Maintaining one instrument across settings standardised the case definition, but CAM-ICU was developed principally for ICU use; among general medicine inpatients, the 3-Minute Diagnostic Interview for CAM-defined Delirium (3D-CAM) has shown greater sensitivity, particularly for milder presentations (19, 35). Ward delirium may therefore have been missed. Assessment ended at discharge; onset day and duration were not recorded, and inter-rater reliability was not assessed. Finally, seven deaths—including the two patients with indeterminate delirium status—permit description only and cannot support a mortality model or causal interpretation.

The study also has strengths. It prospectively screened consecutive elective Bentall candidates over a defined period; scheduled bilateral ONSD at 10 predefined perioperative points, with missing measurements reported explicitly; used separate investigators for sonography, delirium assessment, and cognitive testing; distinguished documented negative outcomes from unassessable outcomes; and reported multiplicity-adjusted time-point comparisons, repeated-measures modelling, penalised regression, internal validation, and four-assignment missing-outcome sensitivity analyses with explicit qualifications. These features improve transparency but do not replace external replication.

## Conclusion

Among the 51 participants with assessable delirium status who underwent elective Bentall surgery, higher perioperative ONSD was associated with documented postoperative delirium. The magnitude and direction of the average ONSD and T6 associations were similar across all four deterministic assignments of the two indeterminate outcomes, but the time-by-group interaction was sensitive to those assignments. No individual time-point comparison survived multiplicity correction, and the T6-minus-Preop change did not provide clear evidence of an association when modelled alone. The results are hypothesis-generating and do not demonstrate intracranial hypertension, cerebral oedema, or a validated delirium threshold. Future multicentre studies should prioritise standardised ophthalmic acquisition, archived images, ONSD/eyeball transverse diameter normalisation, blinded reliability assessment, pre-specified handling of unassessable outcomes, pre-specified baseline and serial models, and external validation against clinically relevant neurological outcomes.

## Data Availability

The raw data supporting the conclusions of this article will be made available by the authors, without undue reservation.
