# Supplementary material for: Association between perioperative optic nerve sheath diameter and postoperative delirium after Bentall surgery: a prospective observational study
Source: Front Med (Lausanne). 2026 Sep 15;13:1940776. doi: 10.3389/fmed.2026.1940776 (PMC13619868; doi:10.3389/fmed.2026.1940776)
Supplement: Supplementary file 1 [file Supplementaryfile1.docx]

STROBE checklist — cohort study

**Manuscript title:** Association Between Perioperative Optic Nerve Sheath Diameter and Postoperative Delirium After Bentall Surgery: A Prospective Observational Study

Final manuscript section, table, and figure locations are provided for each item.

| **Item** | **STROBE recommendation** | Location in manuscript |
| --- | --- | --- |
| Title and abstract |  |  |
| 1a | Indicate the study design with a commonly used term in the title or abstract. | Title (“A Prospective Observational Study”); Abstract, Methods. |
| 1b | Provide an informative and balanced summary of what was done and found. | Structured Abstract, Background–Conclusions, including exploratory status, multiplicity-adjusted findings, internal validation, and limitations. |
| Introduction |  |  |
| 2 | Explain the scientific background and rationale. | Introduction. |
| 3 | State specific objectives, including prespecified hypotheses where applicable. | Introduction (study objectives). |
| Methods |  |  |
| 4 | Present key elements of the study design early. | Materials and Methods, ‘Study design, ethics, and participants.’ |
| 5 | Describe setting, locations, and relevant dates, including recruitment and follow-up. | Materials and Methods, “Study design, ethics, and participants” (single tertiary centre; August 2025–June 2026) and “Delirium and cognitive assessment” (ICU admission to postoperative day 10, hospital discharge, or death). |
| 6a | Give eligibility criteria and the sources and methods of participant selection and follow-up. | Materials and Methods, “Study design, ethics, and participants” and “Delirium and cognitive assessment”; Figure 1. |
| 6b | For matched studies, give matching criteria and numbers of exposed and unexposed participants. | Not applicable; this cohort was not matched. |
| 7 | Clearly define outcomes, exposures, predictors, potential confounders, effect modifiers, and diagnostic criteria. | Materials and Methods, “Outcomes and perioperative data,” “ONSD and cerebral oximetry measurements,” “Delirium and cognitive assessment,” and “Statistical analysis.” |
| 8 | For each variable of interest, give data sources and measurement methods; describe comparability between groups. | Materials and Methods, “Outcomes and perioperative data,” “ONSD and cerebral oximetry measurements,” and “Delirium and cognitive assessment.” The same acquisition and assessment procedures were used for all participants. |
| 9 | Describe efforts to address potential sources of bias. | Consecutive screening; predefined measurement points; separate ONSD, delirium, and MoCA assessors; assessor blinding; Holm adjustment; Firth regression; conditional bootstrap validation; explicit missing-data accounting. Materials and Methods, relevant subsections and “Statistical analysis.” |
| 10 | Explain how study size was determined. | Materials and Methods, “Study design, ethics, and participants”: no a priori calculation; all eligible patients during the predefined 11-month recruitment period were screened. |
| 11 | Explain how quantitative variables were handled and justify groupings or cut-points. | Materials and Methods, “Outcomes and perioperative data” and “Statistical analysis”: continuous summaries and per-unit effects; TCA duration coded as 0 min outside TCA for cohort-wide models; study-defined MoCA decline; internally selected Youden operating point. |
| 12a | Describe all statistical methods, including those used to control for confounding. | Materials and Methods, “Statistical analysis”: Welch/Mann–Whitney/Fisher tests, Friedman/Wilcoxon-Holm analyses, mixed models, Firth models, baseline-adjusted models, and physiological/drug sensitivity models. |
| 12b | Describe methods used to examine subgroups and interactions. | Materials and Methods, “Statistical analysis”: time-by-delirium interaction, TCA-stratified models, and ONSD-by-TCA interaction. |
| 12c | Explain how missing data were addressed. | Materials and Methods, “Delirium and cognitive assessment” and “Statistical analysis”; Results, “Study population”; Figure 1. Two participants never reached a CAM-ICU-assessable state before death and therefore had indeterminate, rather than negative, delirium status. All delirium-dependent analyses used the 51 participants with assessable delirium status (17 documented cases and 34 evaluable non-cases), without imputing an outcome. Outcome-independent descriptive summaries and time-point analyses used all available measurements from the full cohort; the Friedman omnibus tests were restricted to participants with complete serial data (ONSD, n=51; rSO₂, n=52). Other outcome-independent physiological, exposure, and inter-eye analyses likewise retained available observations from the 53-participant cohort. The two indeterminate outcomes were additionally examined with deterministic assignment scenarios (Supplementary Table S11). Other missing measurements and their denominators are reported in the relevant tables and figures. |
| 12d | If applicable, explain how loss to follow-up was addressed. | Follow-up was in hospital through postoperative day 10, discharge, or death; there was no conventional loss to follow-up. Two participants died before ever reaching a valid CAM-ICU assessment, so their delirium status remained indeterminate despite surveillance through death. Their exclusion from outcome-dependent analyses and the accompanying missing-outcome scenarios (Supplementary Table S11) are described under item 12c. Post-extubation ONSD, post-extubation core temperature, time to extubation, and postoperative MoCA were also unavailable for these participants; one additional T4 measurement set was unavailable. |
| 12e | Describe sensitivity analyses. | Materials and Methods, “Statistical analysis”; Results, “Cerebral oximetry and physiological analyses” and missing-outcome analyses; Supplementary Tables S4–S6 and S11. Table S4 reports separate physiological, circulatory-arrest, and drug adjustments; Table S11 reports the primary analysis of participants with assessable delirium status and all four deterministic assignments of the two indeterminate outcomes. |
| Results |  |  |
| 13a | Report numbers of individuals at each stage of the study. | Results, “Study population”; Figure 1: 60 assessed, 55 enrolled and operated, 53 in the full perioperative cohort, 51 with assessable delirium status (34 without documented delirium and 17 with documented delirium), and two with indeterminate delirium status. |
| 13b | Give reasons for non-participation at each stage. | Results, “Study population”; Figure 1. Reasons are reported for the five exclusions before enrolment and two intraoperative exclusions; the two outcome-indeterminate participants remained in the full cohort but had no valid CAM-ICU examination before death. |
| 13c | Consider use of a flow diagram. | Figure 1 shows recruitment, exclusions, the 53-participant full cohort, and the split into 51 participants with assessable delirium status and two participants with indeterminate status. |
| 14a | Give participant characteristics and information on exposures and potential confounders. | Results, “Study population”; Table 1; Supplementary Tables S1 and S5. |
| 14b | Indicate the number of participants with missing data for each variable of interest. | Results, “Study population”; Figure 1; table/figure denominators; Supplementary Tables S1, S6, S7, S9, and S10. Delirium status was assessable in 51/53 participants and indeterminate in two; outcome-independent analyses use available full-cohort measurements. |
| 14c | Summarise follow-up time. | Delirium surveillance window in Materials and Methods; ICU stay and time to discharge or in-hospital death in Table 1 and Supplementary Table S1. |
| 15 | Report numbers of outcome events or summary measures over time. | Results: delirium was documented in 17/51 participants with assessable delirium status (33.3%); 34 had no documented delirium and two further participants had indeterminate status. Motor subtypes, full-cohort serial ONSD/rSO₂ summaries, and 8/51 exploratory MoCA declines are reported. Seven of 53 participants died: five after documented delirium and two while delirium status remained indeterminate. Tables 1–3; Figure 1; Supplementary Tables S1, S6, S9, and S10. |
| 16a | Give unadjusted and adjusted estimates with precision; identify confounders included and explain their selection. | Results, “Baseline, T6, and change-from-baseline analyses” and “Exploratory penalised models and discrimination”; Table 4 and Supplementary Tables S3–S5. Predictor selection is described as data-informed in Materials and Methods. |
| 16b | Report category boundaries when continuous variables were categorised. | The 4.90-mm T6 operating point and its internal selection are reported in Results and Supplementary Table S2B; the exploratory ≥2-point MoCA decline definition is reported in Methods and Supplementary Table S10. |
| 16c | If relevant, consider translating relative risk estimates into absolute risk for a meaningful period. | The absolute documented delirium frequency is 17/51 (33.3%), with two additional indeterminate outcomes. At the internally selected 4.90-mm T6 operating point, the cohort with assessable delirium status yielded TP=10, FN=7, TN=28, and FP=6; PPV was 0.62 and NPV 0.80. The in-hospital observation window and the internal, prevalence-dependent nature of these estimates are reported in Results and Supplementary Table S2B. |
| 17 | Report other analyses, including subgroups, interactions, and sensitivity analyses. | Results, “Perioperative ONSD course and delirium,” “Baseline, T6, and change-from-baseline analyses,” “Cerebral oximetry and physiological analyses,” missing-outcome analyses, and “Inter-eye ONSD concordance”; Supplementary Tables S3–S7 and S11 and Figures S1–S2. Supplementary Table S11 assigns the two indeterminate outcomes in all four possible ways. The mixed-model group estimate and fixed 4.90-mm classification were broadly stable, whereas the time-by-group interaction was sensitive to assignment (p < 0.001–0.094 and non-significant in one configuration); T6 AUC was 0.692–0.743 and the T6 Firth OR was 2.86–4.23. |
| Discussion |  |  |
| 18 | Summarise key results with reference to study objectives. | Discussion. |
| 19 | Discuss limitations, including possible bias or imprecision and their direction or magnitude. | Discussion (limitations). |
| 20 | Give a cautious overall interpretation considering objectives, limitations, multiplicity, and other studies. | Discussion; Conclusion. |
| 21 | Discuss generalisability. | Discussion (strengths and limitations); Conclusion. |
| Other information |  |  |
| 22 | Give the source of funding and the role of funders. | Funding statement: no financial support was received for the research and/or publication. |
