# Supplementary material for: Association between perioperative optic nerve sheath diameter and postoperative delirium after Bentall surgery: a prospective observational study
Source: Front Med (Lausanne). 2026 Sep 15;13:1940776. doi: 10.3389/fmed.2026.1940776 (PMC13619868; doi:10.3389/fmed.2026.1940776)
Supplement: Supplementary file 2 [file Table1.docx]

Supplementary Material

# Supplementary Table S1. Expanded participant and perioperative characteristics by delirium status

| **Domain** | **Variable** | **No delirium (n=34)** | **Delirium (n=17)** | **p value** |
| --- | --- | --- | --- | --- |
| Demographics and comorbidity | Age (years) | 57.3 ± 9.2 | 59.8 ± 10.0 | 0.382 |
|  | Female sex, n (%) | 14 (41%) | 4 (24%) | 0.352 |
|  | Body mass index (kg/m²) | 27.5 ± 3.2 | 27.9 ± 2.5 | 0.643 |
|  | ASA physical status | III: 31 (91%); IV: 3 (9%) | III: 15 (88%); IV: 2 (12%) | 1.000 |
|  | Hypertension, n (%) | 26 (76%) | 10 (59%) | 0.211 |
|  | Diabetes mellitus, n (%) | 3 (9%) | 3 (18%) | 0.387 |
|  | Coronary artery disease, n (%) | 22 (65%) | 9 (53%) | 0.545 |
|  | Carotid Doppler performed, n (%) | 21 (62%) | 9 (53%) | 0.563 |
|  | Previous surgery, n (%) | 22 (65%) | 11 (65%) | 1.000 |
|  | Alcohol use, n (%) | 1 (3%) | 3 (18%) | 0.102 |
|  | Smoking, n (%) | 16 (47%) | 10 (59%) | 0.555 |
|  | Smoking exposure (pack-years) | 0 [0–20] | 10 [0–30] | 0.290 |
|  | Education | Primary: 8 (24%); secondary: 9 (26%); high school: 16 (47%); university: 1 (3%) | Primary: 4 (24%); secondary: 2 (12%); high school: 10 (59%); university: 1 (6%) | 0.590 |
|  | Oral antihypertensive, n (%) | 26 (76%) | 10 (59%) | 0.211 |
|  | Antidiabetic drug or insulin, n (%) | 2 (6%) | 3 (18%) | 0.318 |
|  | Anticoagulant or antiplatelet drug, n (%) | 22 (65%) | 10 (59%) | 0.763 |
|  | Statin, n (%) | 5 (15%) | 5 (29%) | 0.270 |
| Preoperative cardiac and cognitive status | Ejection fraction (%) | 60.0 [50.0–60.0] | 55.0 [50.0–60.0] | 0.279 |
|  | EuroSCORE II | 4.0 [3.0–5.0] | 5.0 [3.0–5.0] | 0.503 |
|  | Preoperative MoCA (points) | 22.8 ± 2.3 | 21.5 ± 1.7 | 0.025 |
|  | Early postoperative MoCA (points) | 22 [21–24] | 22 [21–22] | 0.477 |
| Surgery and perfusion | Operative time (min) | 293.4 ± 73.1 | 323.2 ± 67.4 | 0.157 |
|  | Cardiopulmonary bypass time (min) | 118.0 [102.2–159.2] | 143.0 [124.0–160.0] | 0.204 |
|  | Aortic cross-clamp time (min) | 90.5 ± 38.8 | 101.5 ± 37.7 | 0.338 |
|  | Cerebral perfusion | Antegrade: 34 (100%) | Antegrade: 15 (88%); retrograde: 2 (12%) | 0.107 |
|  | Total circulatory arrest, n (%) | 13 (38%) | 11 (65%) | 0.136 |
|  | Duration of circulatory arrest (min) | 11.0 [9.5–15.0] | 13.0 [11.0–18.5] | 0.172 |
|  | Nadir core temperature (°C) | 31.0 [28.0–32.0] | 28.0 [26.0–30.0] | 0.081 |
| Intraoperative drugs, fluids, and blood products | Methylprednisolone, n (%) | 29 (85%) | 14 (82%) | 1.000 |
|  | Mannitol, n (%) | 26 (76%) | 14 (82%) | 0.731 |
|  | Dobutamine (mg) | 64 [48–104] | 76 [58–125] | 0.147 |
|  | Dopamine (mg) | 55 [7–96] | 75 [0–98] | 0.968 |
|  | Urine output (mL) | 1600 [1315–2062] | 1700 [1300–1800] | 0.772 |
|  | Fluid balance (mL) | 275 [162–650] | 250 [200–500] | 0.856 |
|  | Total intraoperative fentanyl dose (µg) | 600 [512–700] | 600 [600–700] | 0.686 |
|  | Red cells, intraoperative (units) | 1 [0–1] | 1 [0–1] | 0.878 |
|  | Fresh frozen plasma, intraoperative (units) | 2 [2–2] | 2 [2–2] | 0.166 |
| Intraoperative haemodynamic and blood-gas averages | Mean arterial pressure (mmHg) | 74.4 [69.2–78.7] | 76.3 [68.6–77.1] | 0.522 |
|  | Mean PaO₂ (mmHg) | 206.6 [189.4–228.9] | 196.4 [190.7–214.7] | 0.516 |
|  | Mean PaCO₂ (mmHg) | 42.5 [40.2–44.6] | 43.7 [39.1–47.0] | 0.712 |
|  | Mean haematocrit (%) | 34.2 [31.8–36.1] | 34.8 [31.2–36.0] | 0.992 |
|  | Mean lactate (mmol/L) | 2.3 [1.5–2.7] | 2.1 [1.9–2.7] | 0.497 |
|  | Peak lactate (mmol/L) | 3.8 [2.4–5.4] | 3.8 [3.1–5.5] | 0.536 |
|  | Mean pH | 7.39 [7.36–7.41] | 7.37 [7.35–7.39] | 0.194 |
| Postoperative course and outcome | Intensive care unit stay (days) | 2.0 [2.0–3.0] | 3.0 [2.0–3.0] | 0.280 |
|  | Time to extubation (min) | 387.5 [351.2–445.0] | 345.0 [340.0–385.0] | 0.062 |
|  | Time to discharge or in-hospital death (days) | 8.0 [7.0–8.0] | 7.0 [7.0–8.0] | 0.169 |
|  | NRS at 6 h | 3 [3–4] (n=13) | 4 [2.5–4] (n=11) | 0.879 |
|  | NRS at 12 h | 4 [3.25–5] (n=30) | 4 [4–6] (n=17) | 0.661 |
|  | NRS at 24 h | 6 [4–6] (n=33) | 6 [4–6] (n=17) | 0.817 |
|  | Postoperative rescue analgesic dose (mg) | 0 [0–0] | 0 [0–0] | 0.170 |
|  | Mechanical support | None: 30 (88%); pacing: 4 (12%) | None: 12 (71%); pacing: 1 (6%); IABP: 3 (18%); ECMO: 1 (6%) | 0.024 |
|  | Stroke, n (%) | 0 (0%) | 2 (12%) | 0.107 |
|  | Death within 10 days, n (%) | 0 (0%) | 5 (29%) | — |
|  | Red cells, postoperative (units) | 0 [0–0] | 0 [0–1] | 0.065 |
|  | Fresh frozen plasma, postoperative (units) | 0 [0–1] | 1 [0–2] | 0.253 |

Values are mean ± SD, median [IQR], or n (%). Except for NRS, the table and p values include the 51 participants with assessable delirium status (34 without and 17 with documented delirium); the two participants with indeterminate delirium status are excluded. NRS was recorded only in extubated participants able to communicate at the scheduled assessment and therefore uses available cases at 6 h (n=24), 12 h (n=47), and 24 h (n=50). P values are unadjusted and derive from Welch’s t test, Mann–Whitney U test, Fisher’s exact test for 2×2 tables, or the Fisher–Freeman–Halton exact test for larger contingency tables, as appropriate. Circulatory-arrest duration applies to the 24 participants with assessable delirium status who underwent arrest (13 without and 11 with delirium); 26/53 participants underwent arrest in the full cohort. Mortality is descriptive only: no death occurred among the 34 participants without documented delirium, five deaths followed documented delirium, and two additional deaths occurred in the two participants with indeterminate delirium status (7/53 deaths overall); no mortality p value was calculated. ASA, American Society of Anesthesiologists; ECMO, extracorporeal membrane oxygenation; IABP, intra-aortic balloon pump; IQR, interquartile range; MoCA, Montreal Cognitive Assessment; NRS, 0–10 numerical rating scale; PaCO₂, arterial carbon dioxide tension; PaO₂, arterial oxygen tension; SD, standard deviation.

# Supplementary Table S2. Conditional internal validation and internally selected T6 operating point

## Supplementary Table S2A. Conditional bootstrap validation of the exploratory Firth models

| **Model** | **n/events** | **Events per variable** | **Apparent AUC** | **Mean optimism** | **Corrected AUC** | **Calibration slope, apparent → corrected** | **Apparent calibration intercept** | **Apparent Brier score** | **Four-group Hosmer–Lemeshow χ² (df = 2), p** | **Valid/requested bootstrap fits** | **Finite warning fits retained** |
| --- | --- | --- | --- | --- | --- | --- | --- | --- | --- | --- | --- |
| Five-variable model | 51/17 | 3.4 | 0.839 | 0.061 | 0.778 | 1.234 → 0.814 | 0.089 | 0.146 | 1.53, 0.464 | 984/1,000 | 27 |
| T6 ONSD + preoperative MoCA | 51/17 | 8.5 | 0.793 | 0.022 | 0.772 | 1.114 → 1.013 | 0.034 | 0.173 | 5.67, 0.059 | 1,000/1,000 | 0 |
| T6 ONSD alone | 51/17 | 17.0 | 0.728 | −0.001 | 0.728 | 1.091 → 1.253 | 0.044 | 0.187 | 0.97, 0.615 | 1,000/1,000 | 0 |

Harrell optimism correction used patient-level bootstrap resampling with random seed 42 in the 51 participants with assessable delirium status. For the five-variable model, 27 finite fits with convergence warnings were retained and 16 fits failed. The fixed predictor sets were refitted, but the preceding predictor screening and selection of T6 from among the study time points were not repeated; validation is therefore conditional and may remain optimistic. The Hosmer–Lemeshow check has low power in this sample. AUC, area under the receiver operating characteristic curve; MoCA, Montreal Cognitive Assessment; ONSD, optic nerve sheath diameter.

## Supplementary Table S2B. Apparent and bootstrap-corrected performance of the internally selected 4.90-mm T6 ONSD operating point

| **Measure** | **Apparent estimate** | **Uncertainty or validation** |
| --- | --- | --- |
| Selected T6 ONSD operating point (mm) | 4.90 | Bootstrap median 4.90 [4.75–5.10] |
| Sensitivity | 0.59 (0.36–0.78) | Optimism-corrected 0.52 |
| Specificity | 0.82 (0.66–0.92) | Optimism-corrected 0.80 |
| Positive predictive value | 0.62 (0.39–0.82) | Prevalence dependent |
| Negative predictive value | 0.80 (0.64–0.90) | Prevalence dependent |
| Confusion counts | TP 10; FN 7; TN 28; FP 6 | n=51; 17 events |
| Bootstrap 2.5th–97.5th percentile interval (mm) | 4.30–5.30 | 2,000/2,000 valid resamples |

The Youden operating point was selected and evaluated in the same 51-patient cohort with assessable delirium status (17 events). Wilson intervals are shown for sensitivity, specificity, and predictive values. The operating point was reselected in each bootstrap sample, but selection of T6 from among the candidate study time points was not repeated. These internally derived values require external validation and must not be interpreted as a threshold for intracranial hypertension. CI, confidence interval; FN, false negative; FP, false positive; IQR, interquartile range; ONSD, optic nerve sheath diameter; TN, true negative; TP, true positive.

# Supplementary Table S3. Exploratory nested comparisons of ordinary logistic regression models

| **Model comparison** | **n** | **AIC, base model** | **AIC, extended model** | **Likelihood-ratio χ²** | **df** | **p value** |
| --- | --- | --- | --- | --- | --- | --- |
| Preop ONSD; add T6 ONSD | 51 | 66.31 | 62.42 | 5.89 | 1 | 0.015 |
| T0 ONSD; add T6 ONSD | 51 | 65.24 | 62.25 | 4.99 | 1 | 0.025 |
| T6 ONSD; add Preop ONSD | 51 | 60.56 | 62.42 | 0.14 | 1 | 0.711 |
| T6 ONSD; add T5 ONSD | 51 | 60.56 | 62.56 | 0.00 | 1 | 0.999 |

These supportive comparisons used ordinary maximum-likelihood logistic regression and do not establish superiority of a measurement time point. AIC, Akaike information criterion; ONSD, optic nerve sheath diameter.

# Supplementary Table S4. Sensitivity analyses for the association between T6 ONSD and postoperative delirium

## Separate physiological, circulatory-arrest, and drug adjustments

| **Separate Firth model** | **n/events** | **T6 ONSD OR per 1 mm (95% CI)** | **p value** |
| --- | --- | --- | --- |
| Unadjusted | 51/17 | 3.75 (1.44–12.32) | 0.005 |
| Adjusted for PaCO₂ at T6 | 51/17 | 3.62 (1.43–11.50) | 0.005 |
| Adjusted for MAP at T6 | 51/17 | 3.99 (1.41–14.86) | 0.007 |
| Adjusted for haematocrit at T6 | 51/17 | 3.70 (1.44–12.01) | 0.005 |
| Adjusted for pH at T6 | 51/17 | 3.73 (1.43–12.56) | 0.006 |
| Adjusted for core temperature at T6 | 51/17 | 3.52 (1.36–11.31) | 0.008 |
| Adjusted for nadir core temperature at T3 | 51/17 | 3.96 (1.39–15.56) | 0.008 |
| Adjusted for TCA duration | 51/17 | 4.21 (1.48–16.20) | 0.005 |
| Adjusted jointly for PaCO₂, haematocrit, and MAP at T6 | 51/17 | 3.87 (1.43–13.40) | 0.006 |
| Adjusted for TCA exposure | 51/17 | 3.70 (1.38–12.81) | 0.008 |
| Adjusted for methylprednisolone exposure | 51/17 | 3.62 (1.42–11.69) | 0.006 |
| Adjusted for mannitol exposure | 51/17 | 3.62 (1.41–11.78) | 0.006 |

Each row is a separate sparse model in the 51 participants with assessable delirium status. TCA duration was coded as 0 min for the 27 participants without TCA. ORs, profile penalised-likelihood 95% CIs, and penalised likelihood-ratio p values refer to the T6 ONSD term. These analyses cannot exclude residual confounding. CI, confidence interval; MAP, mean arterial pressure; ONSD, optic nerve sheath diameter; OR, odds ratio; PaCO₂, arterial carbon dioxide tension; TCA, total circulatory arrest.

# Supplementary Table S5. Total circulatory arrest strata and protocol-drug records

## Supplementary Table S5A. T6 ONSD association within TCA strata and interaction analysis

| **Model or stratum** | **n/events** | **Term** | **OR (95% CI)** | **p value** |
| --- | --- | --- | --- | --- |
| TCA | 24/11 | T6 ONSD per 1 mm | 3.70 (1.11–22.62) | 0.032 |
| No TCA | 27/6 | T6 ONSD per 1 mm | 3.12 (0.70–18.48) | 0.139 |
| Interaction model | 51/17 | T6 ONSD × TCA | 1.19 (0.14–11.75) | 0.839 |

Estimates are from Firth penalised logistic regression in participants with assessable delirium status. The two participants with indeterminate delirium status both underwent TCA; full-cohort protocol exposure and mannitol-volume summaries remain in Tables S5B–C. CI, confidence interval; ONSD, optic nerve sheath diameter; OR, odds ratio; TCA, total circulatory arrest.

## Supplementary Table S5B. Recorded TCA-protocol drug exposure

| **Drug or protocol exposure** | **No TCA (n=27)** | **TCA (n=26)** | **Overall (n=53)** |
| --- | --- | --- | --- |
| Methylprednisolone, n (%) | 19 (70%) | 26 (100%) | 45 (85%) |
| Mannitol, n (%) | 16 (59%) | 26 (100%) | 42 (79%) |
| Additional thiopental bolus for the TCA protocol, n (%) | Not applicable | 26 (100%) | 26 (49%) |

Administration of the additional thiopental bolus to all 26 TCA patients was verified from the clinical protocol/records by the authors; the analytic dataset had no dedicated patient-level thiopental variable. All participants separately received thiopental for anaesthetic induction as described in the Methods.

## Supplementary Table S5C. Recorded mannitol volumes and additional thiopental-dose availability

| **Drug** | **Stratum** | **Recipients/users, n** | **Recorded volume among users, median [IQR] (range)** |
| --- | --- | --- | --- |
| Mannitol | Overall | 42 | 400 [300–600] (200–800) mL |
|  | TCA | 26 | 400 [325–600] (200–800) mL |
|  | No TCA | 16 | 400 [300–450] (200–600) mL |
| Additional thiopental bolus | TCA | 26 | Dose unavailable |

Mannitol volume is reported in mL exactly as recorded; drug concentration was not encoded and no mass dose was inferred. The patient-level dose of the additional thiopental bolus was not retained in the analytic dataset; the protocol dose is reported in the Methods.

# Supplementary Table S6. Time-aligned physiological values and ONSD correlations

## Supplementary Table S6A. Physiological variables by study time point

| **Variable** | **Preop** | **T0** | **T1** | **T2** | **T3** | **T4** | **T5** | **T6** | **Postop 1 h** | **Post-extubation** |
| --- | --- | --- | --- | --- | --- | --- | --- | --- | --- | --- |
| ONSD (mm), median [IQR] | 4.30 [4.00–4.70] (n=53) | 4.30 [4.00–4.70] (n=53) | 4.40 [4.10–4.80] (n=53) | 4.45 [4.15–5.00] (n=53) | 4.60 [4.10–5.00] (n=53) | 4.55 [4.25–4.81] (n=52) | 4.55 [4.30–4.90] (n=53) | 4.60 [4.30–4.95] (n=53) | 4.50 [4.30–4.90] (n=53) | 4.60 [4.30–4.82] (n=51) |
| Core temperature (°C) | 36.5 ± 0.2 (n=53) | 36.3 ± 0.2 (n=53) | 35.2 ± 0.2 (n=53) | 30.8 ± 1.6 (n=53) | 29.2 ± 2.8 (n=53) | 32.3 ± 0.1 (n=53) | 36.8 ± 0.3 (n=53) | 36.4 ± 0.2 (n=53) | 36.3 ± 0.1 (n=53) | 36.5 ± 0.1 (n=51) |
| PaCO₂ (mmHg) | — | 43.9 ± 6.3 (n=53) | 42.8 ± 6.4 (n=53) | 42.4 ± 5.6 (n=53) | 40.6 ± 7.3 (n=53) | 40.8 ± 7.1 (n=52) | 44.3 ± 7.6 (n=53) | 45.8 ± 7.8 (n=53) | — | — |
| MAP (mmHg) | — | 102 ± 24 (n=53) | 82 ± 14 (n=53) | 66 ± 13 (n=53) | 67 ± 16 (n=53) | 65 ± 15 (n=52) | 67 ± 10 (n=53) | 67 ± 10 (n=53) | — | — |
| Haematocrit (%) | — | 44.3 ± 5.5 (n=53) | 41.8 ± 5.0 (n=53) | 35.3 ± 6.1 (n=53) | 29.5 ± 4.2 (n=53) | 28.1 ± 3.8 (n=52) | 28.3 ± 4.0 (n=53) | 28.5 ± 4.3 (n=53) | — | — |
| pH | — | 7.41 ± 0.05 (n=53) | 7.40 ± 0.05 (n=53) | 7.40 ± 0.04 (n=53) | 7.40 ± 0.07 (n=53) | 7.38 ± 0.08 (n=52) | 7.33 ± 0.08 (n=53) | 7.33 ± 0.08 (n=53) | — | — |
| Lactate (mmol/L) | — | 2.0 ± 1.9 (n=53) | 1.6 ± 0.9 (n=53) | 1.4 ± 0.7 (n=53) | 1.9 ± 1.4 (n=53) | 2.4 ± 1.8 (n=52) | 3.9 ± 3.0 (n=53) | 4.1 ± 3.1 (n=53) | — | — |

ONSD is reported as median [IQR]; physiological variables are mean ± SD. An em dash indicates that the variable was not collected at that point. Core temperature was recorded at all ten study time points. At T4, ONSD, blood-gas variables, and MAP were available in 52 participants, whereas core temperature was available in all 53. Post-extubation ONSD and core temperature were available in 51 participants because two patients were never extubated. Central venous pressure was not entered at the predefined time points and could not be tabulated. IQR, interquartile range; MAP, mean arterial pressure; ONSD, optic nerve sheath diameter; PaCO₂, arterial carbon dioxide tension; SD, standard deviation.

## Supplementary Table S6B. Time-matched correlations between ONSD and physiological variables

| **Physiological variable** | **Time point** | **n** | **Spearman ρ** | **Raw p** | **Holm-adjusted p within variable family** |
| --- | --- | --- | --- | --- | --- |
| Core temperature | Preop | 53 | −0.27 | 0.048 | 0.477 |
|  | T0 | 53 | −0.12 | 0.388 | 1.000 |
|  | T1 | 53 | −0.01 | 0.956 | 1.000 |
|  | T2 | 53 | +0.11 | 0.419 | 1.000 |
|  | T3 | 53 | −0.01 | 0.933 | 1.000 |
|  | T4 | 52 | +0.05 | 0.721 | 1.000 |
|  | T5 | 53 | −0.17 | 0.212 | 1.000 |
|  | T6 | 53 | −0.08 | 0.581 | 1.000 |
|  | Postop 1 h | 53 | −0.09 | 0.525 | 1.000 |
|  | Post-extubation | 51 | −0.18 | 0.217 | 1.000 |
| PaCO₂ | T0 | 53 | +0.02 | 0.878 | 1.000 |
|  | T1 | 53 | +0.07 | 0.642 | 1.000 |
|  | T2 | 53 | +0.19 | 0.182 | 1.000 |
|  | T3 | 53 | −0.07 | 0.600 | 1.000 |
|  | T4 | 52 | +0.21 | 0.136 | 0.950 |
|  | T5 | 53 | +0.03 | 0.859 | 1.000 |
|  | T6 | 53 | −0.03 | 0.849 | 1.000 |
| MAP | T0 | 53 | −0.31 | 0.022 | 0.157 |
|  | T1 | 53 | −0.17 | 0.231 | 1.000 |
|  | T2 | 53 | −0.00 | 0.976 | 1.000 |
|  | T3 | 53 | +0.08 | 0.567 | 1.000 |
|  | T4 | 52 | +0.11 | 0.454 | 1.000 |
|  | T5 | 53 | +0.01 | 0.916 | 1.000 |
|  | T6 | 53 | −0.26 | 0.058 | 0.349 |
| Haematocrit | T0 | 53 | +0.04 | 0.768 | 1.000 |
|  | T1 | 53 | −0.22 | 0.118 | 0.827 |
|  | T2 | 53 | +0.14 | 0.308 | 1.000 |
|  | T3 | 53 | +0.19 | 0.181 | 1.000 |
|  | T4 | 52 | +0.10 | 0.481 | 1.000 |
|  | T5 | 53 | −0.19 | 0.174 | 1.000 |
|  | T6 | 53 | −0.08 | 0.588 | 1.000 |
| pH | T0 | 53 | +0.23 | 0.097 | 0.660 |
|  | T1 | 53 | +0.05 | 0.722 | 1.000 |
|  | T2 | 53 | +0.23 | 0.094 | 0.660 |
|  | T3 | 53 | +0.06 | 0.685 | 1.000 |
|  | T4 | 52 | +0.09 | 0.549 | 1.000 |
|  | T5 | 53 | −0.07 | 0.634 | 1.000 |
|  | T6 | 53 | −0.02 | 0.877 | 1.000 |

Holm adjustment was applied separately within each physiological-variable family. No adjusted p value was below 0.05. ONSD, optic nerve sheath diameter; MAP, mean arterial pressure; PaCO₂, arterial carbon dioxide tension.

# Supplementary Table S7. Right–left ONSD concordance by study time point

| **Time point** | **Paired observations, n** | **ICC(A,1) (95% CI)** |
| --- | --- | --- |
| Preop | 53 | 0.79 (0.66–0.87) |
| T0 | 53 | 0.75 (0.60–0.85) |
| T1 | 53 | 0.78 (0.64–0.86) |
| T2 | 53 | 0.74 (0.59–0.84) |
| T3 | 53 | 0.74 (0.59–0.84) |
| T4 | 52 | 0.58 (0.37–0.73) |
| T5 | 53 | 0.82 (0.70–0.89) |
| T6 | 53 | 0.88 (0.80–0.93) |
| Postop 1 h | 53 | 0.76 (0.62–0.85) |
| Post-extubation | 51 | 0.71 (0.54–0.82) |

ICC(A,1) is a two-way random-effects, absolute-agreement, single-measurement coefficient calculated separately at each study time point to avoid treating repeated occasions within the same patient as independent ICC targets. These coefficients quantify inter-eye concordance, not intra- or inter-observer reliability. CI, confidence interval; ICC, intraclass correlation coefficient; ONSD, optic nerve sheath diameter.

# Supplementary Table S8. Postoperative pain, rescue analgesia, and recorded fentanyl dose by delirium status

| **Variable** | **No documented delirium** | **Documented delirium** | **p value** |
| --- | --- | --- | --- |
| NRS at 6 h | 3 [3–4] (n=13) | 4 [2.5–4] (n=11) | 0.879 |
| NRS at 12 h | 4 [3.25–5] (n=30) | 4 [4–6] (n=17) | 0.661 |
| NRS at 24 h | 6 [4–6] (n=33) | 6 [4–6] (n=17) | 0.817 |
| Postoperative rescue analgesic dose (mg) | 0 [0–0] | 0 [0–0] | 0.170 |
| Total intraoperative fentanyl dose (µg) | 600 [512–700] | 600 [600–700] | 0.686 |

Values are median [IQR]. NRS was recorded only when a participant had been extubated and could communicate at the scheduled assessment; analyses therefore used available cases at 6 h (n=24), 12 h (n=47), and 24 h (n=50). P values are unadjusted Mann–Whitney U tests. All participants received the bilateral parasternal block and scheduled systemic analgesia described in the Methods. These incomplete available-case comparisons do not exclude residual confounding by pain or analgesia. IQR, interquartile range; NRS, 0–10 numerical rating scale.

# Supplementary Table S9. Bilateral regional cerebral oxygen saturation by delirium status

## Supplementary Table S9A. Serial rSO₂ values

| **Time point** | **No delirium, median [IQR] (%)** | **Delirium, median [IQR] (%)** | **Raw p** | **Holm-adjusted p** |
| --- | --- | --- | --- | --- |
| Preop | 64.5 [60.5–71.5] (n=34) | 70.5 [67.5–76.5] (n=17) | 0.103 | 0.618 |
| T0 | 65.0 [60.5–71.4] (n=34) | 68.5 [67.5–76.5] (n=17) | 0.123 | 0.618 |
| T1 | 67.5 [59.5–75.0] (n=34) | 69.0 [66.0–77.0] (n=17) | 0.136 | 0.618 |
| T2 | 60.5 [53.6–67.8] (n=34) | 65.0 [61.0–71.5] (n=17) | 0.025 | 0.201 |
| T3 | 58.2 [50.8–65.6] (n=34) | 61.0 [59.5–69.0] (n=17) | 0.056 | 0.394 |
| T4 | 62.0 [56.1–66.0] (n=34) | 65.0 [58.0–66.0] (n=17) | 0.602 | 1.000 |
| T5 | 67.0 [57.0–72.0] (n=34) | 64.5 [61.0–69.0] (n=17) | 0.992 | 1.000 |
| T6 | 65.5 [58.5–70.4] (n=34) | 68.0 [56.5–73.0] (n=17) | 0.660 | 1.000 |

Between-group comparisons included the 51 participants with assessable delirium status and used Mann–Whitney U tests; Holm adjustment was applied across the eight time points. rSO₂ is the mean of the right and left sensors. IQR, interquartile range; rSO₂, regional cerebral oxygen saturation.

## Supplementary Table S9B. Derived rSO₂ measures

| **Measure** | **No delirium (n=34), median [IQR]** | **Delirium (n=17), median [IQR]** | **Unadjusted p** |
| --- | --- | --- | --- |
| Nadir rSO₂ (%) | 53.2 [47.6–60.5] | 55.5 [52.5–60.0] | 0.363 |
| Maximum decrease from Preop (percentage points) | 11.0 [5.5–14.8] | 10.5 [8.5–16.0] | 0.294 |
| Decrease at T3 from Preop (percentage points) | 6.5 [1.6–12.4] | 7.0 [0.5–11.5] | 0.968 |

These derived comparisons are descriptive and were not included in the eight-time-point Holm family. IQR, interquartile range; rSO₂, regional cerebral oxygen saturation.

# Supplementary Table S10. Complete-case early postoperative MoCA analyses

| **Group** | **n** | **Preoperative MoCA, mean ± SD** | **Postoperative MoCA, mean ± SD** | **Change, median [IQR]** | **Decline ≥2 points, n (%)** |
| --- | --- | --- | --- | --- | --- |
| No delirium | 34 | 22.8 ± 2.3 | 22.6 ± 2.4 | 0.0 [−0.8–0.0] | 6 (18%) |
| Delirium | 17 | 21.5 ± 1.7 | 21.9 ± 1.7 | 0.0 [0.0–1.0] | 2 (12%) |

Paired preoperative and postoperative scores were available in the same 51 participants with assessable delirium status; the overall means were 22.4 ± 2.2 and 22.4 ± 2.2, respectively (paired Wilcoxon p = 0.959). The between-group p value was 0.119 for change and 0.703 for a decline of at least two points (Fisher’s exact test). The two-point threshold was study-defined and exploratory and is not a diagnosis of delayed neurocognitive recovery or postoperative neurocognitive disorder. Postoperative MoCA was unavailable in the two participants who never reached an assessable state before death. IQR, interquartile range; MoCA, Montreal Cognitive Assessment; SD, standard deviation.

# Supplementary Table S11. Sensitivity analyses assigning the two indeterminate delirium outcomes under all four binary configurations

| **Outcome assignment** | **n / events** | **Mixed-model group difference, mm (95% CI)** | **Group p** | **Time-by-group interaction p** | **T6 AUC** | **T6 Firth OR (95% CI)** | **T6 OR p** | **Sensitivity / specificity at 4.90 mm** | **Five-variable apparent AUC** |
| --- | --- | --- | --- | --- | --- | --- | --- | --- | --- |
| Primary: exclude both indeterminate outcomes | 51 / 17 | 0.250 (0.031 to 0.470) | 0.025 | 0.008 | 0.728 | 3.75 (1.44 to 12.32) | 0.005 | 0.588 / 0.824 | 0.839 |
| Both assigned no delirium | 53 / 17 | 0.239 (0.024 to 0.453) | 0.029 | 0.029 | 0.712 | 3.18 (1.31 to 9.22) | 0.010 | 0.588 / 0.806 | 0.796 |
| One assigned delirium and one no delirium (configuration 1) | 53 / 18 | 0.237 (0.026 to 0.448) | 0.028 | 0.094 | 0.692 | 2.86 (1.20 to 7.99) | 0.016 | 0.556 / 0.800 | 0.840 |
| One assigned delirium and one no delirium (configuration 2) | 53 / 18 | 0.248 (0.038 to 0.458) | 0.021 | <0.001 | 0.743 | 4.23 (1.64 to 13.81) | 0.002 | 0.611 / 0.829 | 0.832 |
| Both assigned delirium | 53 / 19 | 0.247 (0.039 to 0.454) | 0.020 | 0.004 | 0.723 | 3.75 (1.49 to 11.68) | 0.004 | 0.579 / 0.824 | 0.856 |

The primary analysis among participants with assessable delirium status excluded the two participants with indeterminate delirium status; the four 53-participant rows are deterministic outcome-assignment scenarios, not observed outcomes. Across assignments, the mixed-model group difference and classification at the fixed 4.90-mm operating point were broadly stable. The time-by-group interaction was sensitive to outcome assignment (p < 0.001–0.094) and was not statistically significant in one configuration. T6 AUC ranged from 0.692 to 0.743, and the T6 Firth OR ranged from 2.86 to 4.23. Five-variable AUCs in this scenario analysis are apparent and were not optimism-corrected. AUC, area under the receiver operating characteristic curve; CI, confidence interval; ONSD, optic nerve sheath diameter; OR, odds ratio.

# Supplementary Figure S1. Repeated-measures agreement between right and left ONSD

Right-minus-left ONSD differences are plotted against the eye mean for 527 paired observations from the full 53-participant cohort. Lines show the mean difference, repeated-measures 95% limits of agreement, and fitted linear slope; the shaded band is the patient-cluster-bootstrap 95% CI for the mean difference. The marginal histogram shows the distribution of right-minus-left differences. ONSD, optic nerve sheath diameter.

# Supplementary Figure S2. Calibration of the exploratory five-variable Firth model

Observed delirium proportions with Wilson 95% CIs are shown for four ascending predicted-risk groups containing 12, 13, 13, and 13 participants with assessable delirium status. The apparent curve and the bootstrap slope-corrected curve are displayed. For plotting, the corrected-curve intercept was re-estimated to preserve the observed event fraction. Predictor screening and selection of T6 from among the study time points were not repeated during bootstrap validation, so the correction is conditional. CI, confidence interval.
